# The impact of a point-of-care testing device on CVD risk assessment completion in New Zealand primary-care practice: A cluster randomised controlled trial and qualitative investigation

**DOI:** 10.1371/journal.pone.0174504

**Published:** 2017-04-19

**Authors:** Sue Wells, Natasha Rafter, Timothy Kenealy, Geoff Herd, Kyle Eggleton, Rose Lightfoot, Kim Arcus, Angela Wadham, Yannan Jiang, Chris Bullen

**Affiliations:** 1 Section of Epidemiology and Biostatistics, School of Population Health, University of Auckland, Auckland, New Zealand; 2 National Institute for Health Innovation, School of Population Health, University of Auckland, Auckland, New Zealand; 3 Department of General Practice and Primary Health Care, School of Population Health, University of Auckland, Auckland, New Zealand; 4 Whangarei Hospital, Northland District Health Board, Whangarei, New Zealand; 5 Te Tai Tokerau Primary Health Organisation, Kaitaia, New Zealand; 6 The National Heart Foundation of New Zealand, Auckland, New Zealand; Public Library of Science, FRANCE

## Abstract

**Objectives:**

To assess the effect of a point of care (POC) device for testing lipids and HbA_1c_ in addition to testing by community laboratory facilities (usual practice) on the completion of cardiovascular disease (CVD) risk assessments in general practice.

**Methods:**

We conducted a pragmatic, cluster randomised controlled trial in 20 New Zealand general practices stratified by size and rurality and randomised to POC device plus usual practice or usual practice alone (controls). Patients aged 35–79 years were eligible if they met national guideline criteria for CVD risk assessment. Data on CVD risk assessments were aggregated using a web-based decision support programme common to each practice. Data entered into the on-line CVD risk assessment form could be saved pending blood test results. The primary outcome was the proportion of completed CVD risk assessments. Qualitative data on practice processes for CVD risk assessment and feasibility of POC testing were collected at the end of the study by interviews and questionnaire. The POC testing was supported by a comprehensive quality assurance programme.

**Results:**

A CVD risk assessment entry was recorded for 7421 patients in 10 POC practices and 6217 patients in 10 control practices; 99.5% of CVD risk assessments had complete data in both groups (adjusted odds ratio 1.02 [95%CI 0.61–1.69]). There were major external influences that affected the trial: including a national performance target for CVD risk assessment and changes to CVD guidelines. All practices had invested in systems and dedicated staff time to identify and follow up patients to completion. However, the POC device was viewed by most as an additional tool rather than as an opportunity to review practice work flow and leverage the immediate test results for patient education and CVD risk management discussions. Shortly after commencement, the trial was halted due to a change in the HbA_1c_ test assay performance. The trial restarted after the manufacturing issue was rectified but this affected the end use of the device.

**Conclusions:**

Performance incentives and external influences were more powerful modifiers of practice behaviours than the POC device in relation to CVD risk assessment completion. The promise of combining risk assessment, communication and management within one consultation was not realised. With shifts in policy focus, the utility of POC devices for patient engagement in CVD preventive care may be demonstrated if fully integrated into the clinical setting.

**Trial registration:**

Australian New Zealand Clinical Trials Registry ACTRN12613000607774

## Introduction

Diabetes and cardiovascular disease (CVD) are major causes of death and disability in New Zealand[1,2] with Māori (indigenous people of New Zealand), Pacific and South Asian people bearing a disproportionate burden.[3,4] To address this population health issue, in 2011, the government made CVD risk assessments and screening for diabetes a national priority, setting a target of 90% of eligible adults to be screened by Primary Health Organisations (PHOs) by July 2014.[5] According to CVD risk assessment performance, PHOs would receive modest incentive payments and would be benchmarked quarterly against others.[6]

New Zealand general practices typically comprise small groups of doctors and nurses co-located in shared facilities sometimes with allied health services such as physiotherapists. Each practice belongs to one of 32 PHOs that receive government capitation funding for their enrolled populations. In addition, patients usually pay a co-payment of around $45–50 per visit ($17 for disadvantaged groups).

As part of a CVD risk assessment, national guidelines recommend blood lipid testing and use of the ratio of total cholesterol to high density lipoprotein (TC/HDL) in the risk prediction algorithm.[7] In 2009, screening for diabetes was also recommended as part of the risk assessment using either fasting glucose or glycated haemoglobin (HbA_1c_). The uptake of screening tests has been reported as being more of a problem in rural areas, for high risk ethnic groups and for those who are most socio-economically deprived.[8–13]

A new point-of-care (POC) device (cobas b 101, Roche Diagnostics International Ltd) that can perform both TC/HDL and HbA_1c_ tests became available on the New Zealand and global markets in 2013. In the context of CVD risk assessment, use of the device in general practice would relieve the burden for patients to have to travel to a community laboratory, often located a considerable distance away from their home and GP’s offices, for blood testing. It would also enable clinicians to get near immediate test results and commence discussions on preventive care. Primary care colleagues (KE, RL) estimated this could be achieved in a 30 minute consultation (including 15 minutes to undertake the finger prick sampling and provide the test results).

However, its use in NZ general practice settings had not been evaluated and, typically, GPs and practice nurses have 15 minute appointments. We aimed to assess the effect of the POC device on the frequency of completed CVD risk assessments in general practices in the Northland region of New Zealand and whether the introduction of this technology would impact on ethnic disparities in screening. This region has two PHOs which collaborate on population health strategies and information services and serve 170,000 people with two-thirds living in rural communities. Compared to the national average, the population is significantly older and has a much higher proportion of Māori and people living in the poorest areas of socio-economic deprivation.[14]

## Methods

### Study design

The EPOCH (Evaluating a Point of Care device in Heart healthcare) trial is a pragmatic, cluster randomised controlled trial in general practice. We incorporated a practice interview and questionnaire at the end of the trial to assist in interpreting the findings. The outcome of interest was completion of CVD risk assessments. Intervention practices were trained to use a cobas b 101 POC device in addition to usual care and the control practices conducted usual care alone.

### Setting and participants

All 38 general practices in the Northland region were invited to participate in the trial. Patients enrolled in these practices were included if they were 35–79 years old, had no previous history of CVD (including previous stroke, angina, myocardial infarction, peripheral vascular disease, cardiac bypass or other coronary or arterial procedures) and met national guideline criteria for an initial CVD risk assessment or follow-up assessment[7], as determined by their GP. The recommended frequency of follow-up CVD risk assessments varies from five yearly to annually (people with diabetes, those previously found to have high CVD risk [5-year CVD risk ≥15%], or people taking CVD medications).

Practices were randomised centrally by a University of Auckland biostatistician using a computer-generated list, with stratification by location (rural vs urban) and size of practice (small, [< two full time equivalent {FTE} doctors], or large [≥2 FTE doctors]). Study investigators, practice and PHO staff were unable to intervene in the randomisation procedure or allocation. At the end of the trial, the control practices were offered a POC device if desired.

### Sample size

Sample size calculations (standard two-sample t-test with normal approximation for a binomial distribution) drew on estimates of the number of people eligible for a CVD risk assessment (first or a follow-up assessment) and projections of regional CVD risk assessments for a baseline rate of 70% in the control group (personal communication, C Wiltshire, Enigma Solutions Ltd) and an effect size of 15% (an absolute increase to 85%) in the POC group. The effect size estimate was based on a study set in Canadian community pharmacies where POC testing for cholesterol risk management led to a 10% increase.[15] Assuming an intraclass correlation coefficient of 0.025 (from three New Zealand cluster randomized controlled trials[16]) a 5% two-sided level of significant test and power of 90%, we estimated 1000 individual patient risk assessments would be required (on average 50 patients per practice) to establish superiority of the intervention over control conditions.

### Intervention training and quality assurance

We implemented a comprehensive general practice internal quality control programme (IQC) according to the requirements of the ISO 22870: 2006(E) standard and the NZ Best Practice Guidelines for Point-of-care Testing[17] together with external quality assurance(EQA) from two New Zealand laboratories (Canterbury Health Laboratory [CHL] and Northland District Health Board Laboratory [NDHBL]). Both laboratories are accredited by International Accreditation New Zealand on the medical testing standards ISO 15189: 2012 and ISO 22870:2006(E) for POC testing. Members of each general practice team who would use the cobas b 101 device were trained by staff from Roche Diagnostics NZ Ltd for approximately 1.5 to 2 hours on finger prick testing, capillary sampling, use of the device, routine maintenance and ongoing quality requirements. IQC included paired testing of samples to ensure that staff were achieving results on the POC device comparable to standard laboratory testing. The cobas b 101 machine was electronically linked to the practice computer systems to transfer results directly to the patient’s own electronic health record. General practice staff were instructed to use the POC device solely for CVD risk assessments (and not for monitoring of glycaemic control or lipids).

### Technical problems

Prior to commencing the trial, CHL tested several batches of cobas discs against laboratory analysers and found results of acceptable standard (unpublished data). EPOCH began on 12 August 2013 with practice training. However, on 29 August 2013 the trial was halted following an assay precision problem identified with the batch of cobas b 101 HbA_1c_ discs being used in the trial. The investigators notified all practices to stop using the POC device and informed the ethics committee. The 22 individuals who had been tested for HbA_1c_ with the POC device were advised by their practices to have a laboratory HbA_1c_ test. Roche Diagnostics International Ltd instituted a global recall and identified a manufacturing fault. Three months later, a new batch of HbA_1c_ discs became available. This batch was then validated in two independent New Zealand laboratories (LabTests Ltd and CHL). On the basis of these results and data from NDHBL, it was determined that it was safe to resume the trial following retraining and recertification of the 10 practices. After ethical approval, the trial restarted in May 2014. The practices were required to continue monthly paired, IQC tests using two levels of the manufacturer’s quality control solutions. In addition three EQA survey samples with known concentrations of HbA_1c_ and lipids were distributed by NDHBL for analysis on the POC device at each practice over the course of the trial and three additional EQA surveys occurred for external accreditation. The results confirmed good performance of the Roche cobas b101 system using Clinical Laboratory Standards Institute EP-5 and EP-9 protocols.[18] The IQC data showed CV levels of 5.2% and 4.2% for the level 1 and level 2 quality control solutions. Reference laboratory CV’s for level 1 and level 2 quality control solutions at different HbA_1c_ levels varied between 2.0% and 4.9%.

However, by the time the trial restarted, the two PHOs in Northland had documented CVD risk assessment completions for approximately 80% of their eligible enrolled populations (personal communication C Wiltshire, Enigma Solutions Ltd.). Therefore we revised the analysis plan and adopted a non-inferiority hypothesis (that the proportion of CVD risk assessment completions in the POC practices would be no less than that in the control practices) at the restart. Our revised sample size was much the same as previously calculated.

### Outcomes and follow-up

The primary outcome was a completed CVD risk assessment conducted for eligible patients in the 20 participating practices. A completed risk assessment was defined as containing all co-variates necessary to calculate a CVD risk assessment score and to screen for diabetes. Two study periods were defined: eight months prior to the trial period (01 September 2013 to 30 April 2014) and 12 months after intervention (01 May 2014 to 30 June 2015). Secondary outcome measures included; incomplete CVD risk assessment that had been electronically saved (e.g. awaiting patient CVD risk factor data such as blood tests); and time (in days) to complete CVD risk assessment. We measured differences in completion rate by practice location (urban/rural), practice size (large/small), age group, gender, Māori/non Māori, and deprivation (NZDep) quintile. The NZDep Index Score, a measure assigned to a patient’s area of residence based on nine variables from the Census reflecting eight dimensions of relative deprivation of census tracts,[19] ranges from 1 to 10, but for these analyses they were aggregated into quintiles (1 to 5, from least to most deprived).

### Data collection

All practices in Northland have a web-based CVD risk assessment and management decision support system (called “PREDICT”) integrated within their electronic health records (EHR).[20,21] When a GP or practice nurse opens a PREDICT on-line form within a patient’s EHR, the software automatically pulls in relevant clinical and demographic data from the EHR. This can then be checked and any missing or incorrect fields can be updated by the clinician. The following variables are required fields for calculating a patient’s 5-year CVD risk: date of birth, sex, ethnicity, prior diagnosis of CVD, prior diagnosis of diabetes (type 1, type 2 or type unknown), atrial fibrillation, a self-reported family history of premature ischaemic CVD, smoking status, systolic and diastolic blood pressure (mean of two measures), and TC/HDL (one measure). Ethnicity was defined according to a national prioritisation protocol[22] in the following order; Māori, Pacific, Indian and NZ European combined with other ethnicities (Euro/Other). Family history of premature CVD was defined as a self-reported familial history of ischaemic heart disease or ischaemic stroke occurring in a father or brother before 55 years of age, or a mother or sister before 65 years of age. Screening for diabetes (either HbA_1c_ or fasting glucose was made a compulsory field after national guideline updates in 2009.[23] Practices with the POC device could either use the device or the community laboratory to provide TC/HDL or HbA_1c_ results for their patients.

Once a completed form is submitted by the clinician to the web server, a risk score is computed and returned to the clinician. This score and the patient risk profile are stored both in the EHR and on the centrally hosted secure webserver. If screening blood tests or other patient data are not available the assessment can be ‘parked‘(i.e. saved on the webserver and in the EHR until all data are subsequently available).

### Blinding

While it was not possible to blind practices to having the POC device or not, all study investigators (except GH) were blinded to the practice allocation until the end of the trial when one investigator visited the practices, interviewed staff and administered the questionnaire. GH developed and supervised the quality assurance programme, set QA testing protocols and fed back results to practices. Other than this study staff had no influence, or involvement in, how the device was used in the practice, or the collection or recording of clinical data. All study data collection was conducted through the PREDICT programme as part of routine clinical practice. At the end of the trial de-identified data from complete and incomplete assessments from the participating practices were extracted and sent to the investigators for analyses.

### Practice interviews and questionnaires

Two short questionnaires were designed for intervention and control practices to assess current CVD risk assessment procedures, resources for blood testing within practices and the real (intervention) or expected (control) experience of POC testing. At the end of the trial, one investigator (SW) conducted face-to-face interviews or telephone calls to administer the POC- or control-specific questionnaires with the key person/s who led the CVD risk assessment performance in each practice Informant comments were written down and checked for accuracy and completeness with the participants at the time of the interview. Data were de-identified to ensure confidentiality. Braun and Clarke’s approach to thematic analysis was used to generate initial codes, collate codes into potential themes and refine the identified themes and categories into a coherent patterm.[24]

### Statistical analysis

Statistical analyses were performed on the intention to treat principle using SAS version 9.4 (SAS Institute Inc., Cary, NC, USA). All statistical tests were two-tailed at the 5% significance level. Continuous variables were presented as mean and standard deviation with categorical variables presented as frequency and percentage. Generalized linear mixed models were used in treatment evaluations, adjusting for the random effect of clustering and important baseline prognostic factors. The point estimate and 95% confidence interval were reported as appropriate to the link function for a continuous (identity) or categorical (logit) outcome variable. The intra cluster correlation (ICC) coefficient was calculated on the primary outcomes using the observed trial data.

We have reported the RCT component of the study according to the CONSORT statement, and have included the qualitative results so as to provide a richer understanding of the important contextual factors.[25]

### Ethics statement

Written consent was obtained by all participating practices. The EPOCH study was initially approved by the Northern B Health and Disability Ethics Committee (Reference 13/NTB/79). The committee was informed of the trial being halted in August 2013 and subsequently gave approval to recommence the trial in April 2014.

## Results

**Fig 1:**
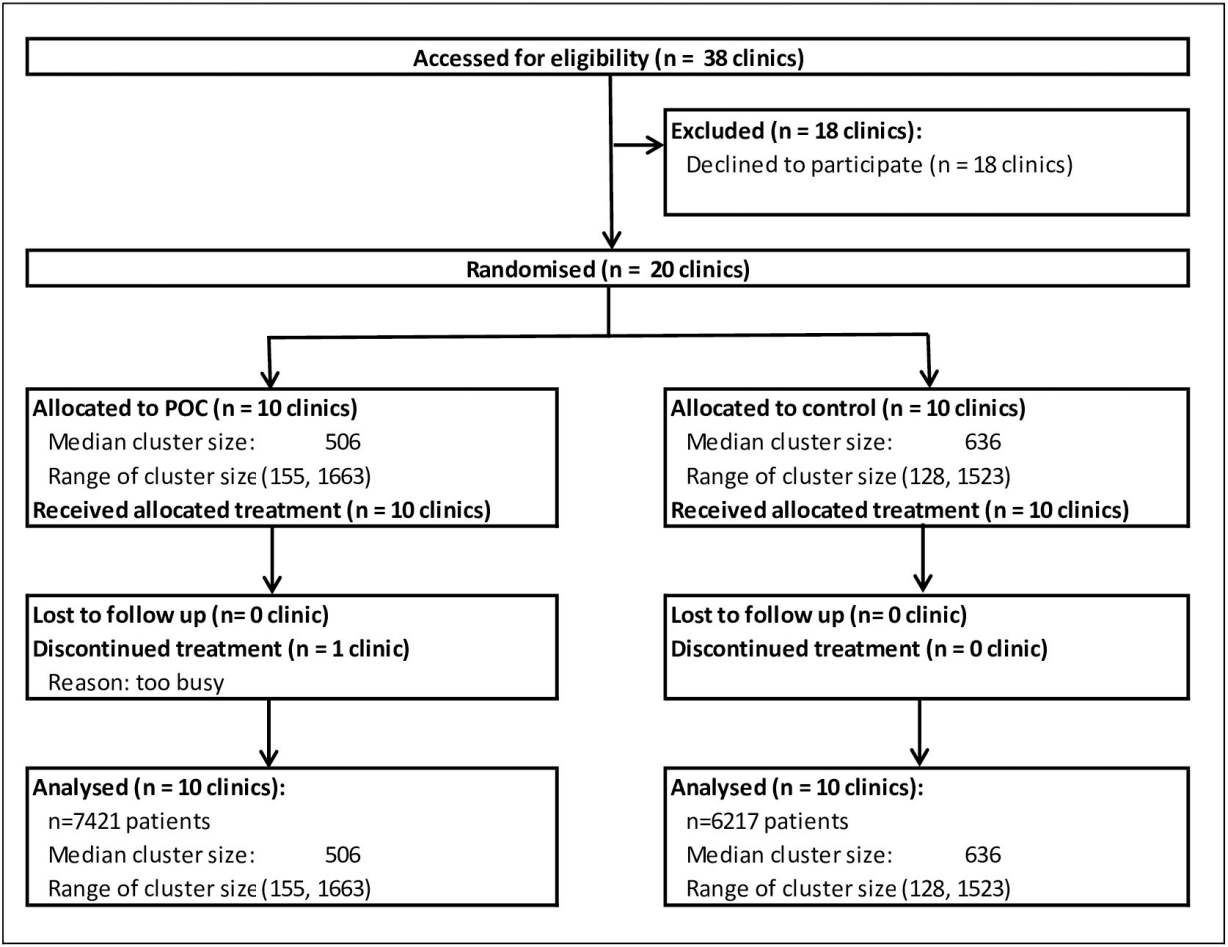
Consort diagram of participation.

Of the 38 practices in the Northland region, 20 (53%) agreed to participate and were randomised. There were 13 rural practices outside of the main city (Whangarei) boundary and 13 practices were classified as being large (≥2 FTE doctors). Stratified randomisation ensured a balance of these characteristics with 6 control and 7 POC practices classified as being rural and 6 control and 7 POC practices classified as being large. Fig 1 shows the CONSORT diagram of participation. One practice discontinued the use of the cobas b 101 device but as all CVD risk assessments were captured electronically regardless of device use, all their data were included in the intervention group data at the end of the trial.

In the 12 months of follow-up after trial commencement, a PREDICT CVD risk assessment entry was recorded for 6217 patients from the control practices and 7421 patients in the POC practices—equivalent to an average of 52 and 62 individual patient entries per practice per month respectively (Table 1) This average monthly rate of CVD risk assessment entry post-intervention was the same as the pre-intervention months.

**Table 1:** Characteristics of patients who had an on-line CVD risk assessment form opened in the 8 months before and after 12 months commencement of the trial.

	Before Trial (Sep 2013 –Apr 2014)				Trial Period (May 2014 –Jun 2015)			
	Control (N = 4090)		POC (N = 4965)		Control (N = 6217)		POC (N = 7421)	
	n	%	N	%	n	%	N	%
Female	1859	45.5	2258	45.5	2806	45.1	3346	45.1
Ethnicity								
Māori	1210	29.6	1681	33.9	1866	30.0	2521	34.0
Pacific	56	1.4	56	1.1	109	1.8	113	1.5
Indian	18	0.4	27	0.5	40	0.6	46	0.6
Other	2806	68.6	3201	64.5	4202	67.6	4741	63.9
Age group (years)								
35–44	300	7.3	383	7.7	475	7.6	651	8.8
45–54	1031	25.2	1475	29.7	1583	25.5	2036	27.4
55–64	1360	33.3	1717	34.6	2127	34.2	2483	33.5
65–74	1256	30.7	1226	24.7	1724	27.7	1919	25.9
75–79	143	3.5	164	3.3	308	5.0	332	4.5
Mean age (std)	59.2 (10.0)		57.8 (9.8)		58.9 (10.2)		58.2 (10.2)	
NZDep quintile^a^								
1	222	5.4	407	8.2	411	6.6	538	7.3
2	436	10.7	676	13.6	714	11.5	993	13.4
3	954	23.3	983	19.8	1445	23.2	1382	18.6
4	1146	28.0	1144	23.0	1773	28.5	1918	25.9
5	1259	30.8	1660	33.4	1771	28.5	2386	32.2
Family history CVD	877	21.4	901	18.2	1230	19.8	1317	17.8
Current smoker	777	19.0	1086	21.9	1273	20.5	1662	22.4
Diabetes	805	19.7	854	17.2	1225	19.7	1385	18.7

^a^NZDep Missing data on 168 patients before trial and 307 patients during the trial as not a compulsory field for CVD risk assessment

Table 1 shows the characteristics of the patients who had a CVD risk assessment on-line form opened (either ‘parked’ or completed) over the 8 months prior and 12 months follow-up post randomisation. About one third of CVD risk assessments entries were recorded for Māori and over 50% of CVD risk assessments were on patients living in the most deprived areas of deprivation (quintiles 4 and 5).

### Main outcomes

Having a POC testing device within the practices made no difference to the completion of CVD risk assessments. During the trial period 99.5% of CVD risk assessment templates in both groups had complete data (Table 2). Furthermore, given that over 99% of the CVD risk assessments were completed on the same day, there were no statistically significant differences in the other secondary outcomes (incomplete CVD risk assessments, time [in days] to complete CVD risk assessment or differences by size of practice or rurality or by patient characteristics).

**Table 2:** Data completion in CVD risk assessments in the 12 months post randomisation.

Completed CVD assessment		Treatment Group			
		Control		POC	
		n	%	n	%
All Practices					
Overall	TOTAL	6217	100.00	7421	100.00
	No^a^	30	0.48	36	0.49
	Yes^b^	6187	99.52	7385	99.51
Practice by size					
Large	TOTAL	5184	83.38	6798	91.60
	No	22	0.42	34	0.50
	Yes	5162	99.58	6764	99.50
					
Small	TOTAL	1033	16.62	623	8.40
	No	8	0.77	2	0.32
	Yes	1025	99.23	621	99.68
Practice location					
Rural	TOTAL	3894	62.63	5168	69.64
	No	15	0.39	23	0.45
	Yes	3879	99.61	5145	99.55
					
Urban	TOTAL	2323	37.37	2253	30.36
	No	15	0.65	13	0.58
	Yes	2308	99.35	2240	99.42

^**a**^ Yes—completed individual patient CVD risk assessment with all data present (age, gender, ethnicity, history of CVD, diabetes diagnosis, family history, smoking status, BP, TC/HDL and either HbA_1c_ or fasting glucose).

^b^No–incomplete or “parked” CVD risk assessment- one of more data elements missing.

Important subgroup factors (age, gender, ethnicity, history of CVD, diabetes diagnosis, family history, smoking status, BP, TC/HDL and either HbA_1c_ or fasting glucose) were investigated in all regression analyses. As only age per year and diabetes status were significantly associated with the primary outcome, the remaining co-variates were removed from the final model. After adjusting for age in years (adjusted odds ratio OR 1.05 95% CI 1.03–1.08) and diabetes status (adjusted odds ratio OR 12.57 95% CI 1.74–90.87), there were no significant differences in CVD risk assessment completion between the two groups (adjusted odds ratio 1.02; 95% CI 0.61–1.69). The estimated ICC coefficient was 0.002, so any potential cluster effect was considered negligible. Of the CVD risk assessments in the intervention practices, 98% were completed using community laboratory values, 2% (124/7421) by POC device. This is not surprising given blood tests up to five years old in a patient’s electronic record could be used to complete a risk assessment entry. In terms of device use, 94% were completed by large practices and 78% in rural clinics.

### Questionnaire and interview findings

Practice interviews took place at the end of the trial, 13 face-to-face and six via telephone call. There were 10 informants (eight practice nurses, one practice manager and one practice assistant) from the nine POC practices and 10 informants (nine practice nurses, one practice manager) from the 10 control practices. Three overall themes emerged; i) the importance of context, specifically usual policy, procedures, staff time and resources ii) *expected* usefulness of the POC device from the control group versus *actual* ease of use and usefulness from POC group and iii) how POC testing might fit into practices in the future.

### The context of CVD risk assessment in both POC and control practices

In this study involving 19 practices, 90% of the eligible patients had already had a CVD risk assessment by the time the POC device was re-introduced.[6] The emphasis was on undertaking a CVD risk assessment for all patients in the practice population within five years, rather than ensuring population groups with high CVD risk had the recommended frequency of monitoring. In most practices, patients with long term conditions or multi-morbidity were being seen at no charge (due to PHO-funded initiatives) by the practice nurse and blood tests arranged prior to or at the time of the visit. In addition, four out of nine POC and five out of 10 control practices routinely collected blood samples on their patients on-site.

One of the major barriers for practices to reach their targets for CVD risk assessments was difficulty getting the last 10% of eligible people to attend. If these patients did come in they often required a full set of laboratory investigations, not just HbA_1c_ and TC/HDL. The time taken to perform venepuncture (intervention and control practices) was estimated as being around 15 minutes—similar to that reported by the practices for use of the cobas b 101 machine.

### Expected usefulness of POC device for CVD risk assessment (control practices)

Many (8/10) of the control nurses were keen to use the POC device once the trial was completed.

“Especially if people can't get to the laboratory. Catch them while they are in the practice. Hard to reach group especially. At the moment patient goes to laboratory, has blood taken, fax / report results, then nurses have to ring on the phone and it takes a lot of time. If patient has POC test, they are right there in front of you. All done and dusted". (Control Practice nurse)

“rather than nurses spending two hours in the afternoon trying to contact patients with blood test results—then unsure if patient understands what the nurse is saying. Much quicker and then finished face to face.” (Control Practice nurse)

### Actual ease of use and usefulness (POC practices)

The most common concern (8/10 POC respondents) about ease of use was that POC testing was not a good use of their time. Work flow changed as a result of having the device as nurses needed to get a sample at the start of a patient consultation. Nurses reported feeling so time-pressured with their workload that having to wait by the machine and the sequential nature of the analysing (one test taking five minutes then the next test taking five minutes) was a negative aspect they had not anticipated.

“It was the waiting 5 minutes—we can do a lot of things in 5 minutes but we were tied to the machine” (POC Practice nurse)

Additional time was required to conduct the monthly quality assurance measures that, if not done, would lock them out from further testing. The other practical limitation for practices was finding a space for the POC device. Usually it was kept in one of the nurse’s consultation rooms which precluded others from using it. The POC device was seen as an additional tool rather than an opportunity to review practice work flow and systems for CVD risk assessment. The present time was more highly valued than the future time gained by the immediacy of getting a result and starting discussions on heart health. Follow-up with patients (from both POC and control groups) to inform them about their laboratory blood tests results and CVD risk assessment typically would take an additional 15–30 minutes depending on the patient CVD risk profile.

The two intervention practices that found the device most useful had purposely changed their systems and allocated extra staff time and space for the POC testing.

“We decided to run a nurse clinic with dedicated time…. This worked much better than fitting things in opportunistically- working on the floor nurses are stressed and stretched and the time it took to do the cobas test was really disruptive.”” (POC Practice nurse)

Several nurses commented that opportunistic screening using POC testing would be most useful for younger people (e.g. <40 years) and men.

“particularly younger ones, 45-55yrs, Māori men loved it—instant result” (POC Practice nurse)

Seven out of ten respondents volunteered that the finger prick was better from a patient perspective than venepuncture and seemed to smooth the path to engagement.

“its good for some people who are hard to get to the clinic- we use it as a first point of engagement—after that we would take full bloods” (POC Practice nurse)

Furthermore the ability to get results almost immediately meant that discussion about the meaning of the CVD risk score could happen without delay.

“We loved the tool as we got results back immediately and so could start the conversation” (POC Practice nurse)

### Place of POC testing for HbA_1c_ and TC/HDL in the future

Most practices (8/10 control and 7/9 POC) indicated that the place of POC testing in the practice was monitoring (rather than screening), especially for patients with diabetes in nurse- led chronic disease clinics. However, the majority of respondents (7/9 POC practices and 5/10 control practices) reported that they would not conduct POC testing if the cost of the consumables (the lipid and HbA_1c_ discs) was borne by the practice.

## Discussion

Having a POC device within NZ general practices made no discernible difference to the completion of CVD risk assessments and was neither superior nor inferior to usual practice. In addition we found no differences in completion by patient characteristics (such as ethnicity or deprivation). However, the context to this trial is important in interpreting these findings. Driven by the government target to undertake CVD risk assessments, practice staff had accelerated their systems and procedures so that 50–60 CVD risk assessments were being conducted on average per month per practice. Indeed by July 2014 (three months after the trial recommenced), both trial and non-trial practices in the region had conducted a CVD risk assessment in 90% of the eligible Northland population in the previous five years [6]. Therefore there was a considerable ceiling effect. Benefits from interventions are more likely to be reported where there is a sizeable evidence-practice gap at baseline. Even if this trial had not been halted it is debatable whether we might have seen a difference given the enormous momentum generated from the national health target. Associated with other external pressures such as open benchmarking, the target was a powerful modifier of practice behaviours. Each practice had responded by extensive investment in staff time and resources with the whole practice team concentrating on getting their eligible adult population risk assessed.

It has been widely recognised that initiatives such as public reporting, pay-for-performance and the introduction of health targets, while well-meaning and seeking to improve health care quality, often have unintended negative consequences.[26–28] One such consequence is ‘tunnel vision’—whole pathways of care being neglected due to focus on one aspect.[29] A further external influence was a guideline update on CVD risk assessment published in late 2013 (while the trial was halted).[30] This update included a new section on ‘non-face-to-face CVD risk assessments’ which made it acceptable to complete an assessment *without* seeing the patient using lab results previously recorded in the electronic health record.[30] Anecdotally in many general practices, the focus on CVD risk assessment has resulted in a backload of work required for adequate CVD risk communication, shared decision making and CVD risk management.

It was unfortunate to halt the trial within weeks of commencing owing to a change in the assay performance of the HbA_1c_ test discs, which was outside the control of the investigators. Practices were then disengaged and there was a loss of confidence that was hard-won to get back. Despite this there were some important learnings. Firstly this was the first dual testing kit available in New Zealand with both the TC/HDL and HbA_1c_ results. The authors believe the experience of this trial is important because POC devices are not comprehensively regulated in New Zealand or in many other jurisdictions.[31] We would recommend that all POC devices introduced to the market for patient care are subject to appropriate validation, quality assurance and be evaluated in populations for whom it is indicated.[32]

Commonly cited disadvantages for POC testing include the lack of training, poor standardization in obtaining blood samples and insufficient internal/external quality assessment.[33] The quality management processes which were designed to support the EPOCH trial were a key factor in the identification of the HbA_1c_ disc problems and also to assure the EPOCH Steering Group that the trial could be restarted once the problem had been rectified. The paired testing monthly quality control conducted by practices in the trial showed that results reasonably close to laboratory standards could be obtained from 9 analysers used by different operators at the 9 different practice locations. For HbA_1c_, the use within a screening programme is especially challenging because two cut-points are required for clinical management decisions; 40 mmol/mol or below indicates normal glucose tolerance and 50 mmol/mol or above indicates diabetes (if confirmed).[23]

The potential of a POC testing device within a practice to change the nursing model of care and therapeutic turn-around-time was widely recognised by respondents—that a point of care test in the practice could facilitate the next steps (CVD risk and risk factor communication) and create an opening to commence conversations about lifestyle changes and medication management. Nurses noted that they spent hours trying to contact people about their laboratory test results and then were uncertain about patient understanding. While POC devices offer greater convenience to patients and more timely clinical decision making,[34] as highlighted by two of the intervention practices, integration of the tool into practice workflow is necessary to derive value for nurses and patients. Like all new technologies introduced into a work place, perceived usefulness and perceived ease of use are hypothesized to be fundamental determinants of user acceptance.[35] The benefits must outweigh the time and effort required to use the new technology in order to be sustainable.[35] The nurses in our trial immediately identified the usefulness for monitoring of people with diabetes as nurse-led clinics for long-term conditions are already embedded in practice culture. For opportunistic screening, the barriers of time, space and cost were seen as too high. If the POC results were delivered faster or concurrently then practices may have seen more value in the device. As it took 15–30 minutes (to get a result and to deliver advice), it was deemed to be no different for staff time than sending someone off to the laboratory for a test and following up at another time.

From published literature we could only find trials investigating the use of POC testing for monitoring patients with established diabetes or hyperlipidaemia[36,37] (as opposed to *screening* for diabetes or CVD risk assessment). Only one trial found the influence of POC testing on glycaemic control to be the same or better than laboratory testing,[38] was associated with same or better medication adherence[37] increased patient satisfaction in general practice[39] significant cost savings to patients and families[40], but cost-effectiveness was uncertain.[40] The latter outcome is pertinent to our study. While the discs and IQC material were supplied free of charge for this trial they would not be in routine care and central laboratories are able to perform assays at a lower cost per analysis due to economies of scale. Therefore to set up a screening programme using POC testing would need to take into account the cost of buying a POC test device for every practice and the on-going cost of the consumables. It is unlikely that general practices themselves would use the machines if they were required to pay for the testing consumables. Furthermore, if the cost was passed onto patients then it is unlikely that socio-economically disadvantaged people would pay for it and this might potentially increase in disparities in CVD risk management. However, the higher cost for POC testing needs to be balanced alongside the opportunity for improved engagement with patients, which may in turn improve motivation and behavioural lifestyle change.

Randomised controlled trials (RCTs) are considered to be the optimal study design to test whether an intervention works and to what extent[41] although this has been challenged as being inadequate within the complexities of real-world research.[25,42] Introducing a POC device into practice is a complex intervention and as such poses major challenges to the RCT design as it is largely determined by human agency.[43] It could be argued that this multifaceted intervention may have been better if we provided more training and support to embed such a tool into practice workflow. However, this approach might lead to difficulties identifying which components were needed to ensure success. The qualitative components provided a deeper understanding of the data [44]. Such a mixed methods approach allows ‘multiple ways of knowing’ [45] as the negative trial results alone do not provide meaning and context of the intervention.

Future evaluation underpinned by a ‘realist’ philosophy[46] would be of great use to policy makers and PHOs. In other words, to determine the place of POC devices in the CVD prevention and management pathway we need to know in what circumstances would POC testing work best, for which group of patients and why.[46] For example, the particular value of POC testing for young, Māori men was highlighted by some respondents. Others indicated that face-to-face communication of health messages is fundamentally different to that delivered over the phone or by letter, with health literacy and understanding of the result enhanced by the immediacy of POC testing. To have real impact on population health, this might be the most important outcome to evaluate into the future.

### Conclusion

The introduction of a POC testing device in urban and rural New Zealand general practices was not associated with increased completion of electronic CVD risk assessment templates. An external policy change exerted a far greater influence on practitioner behaviour.

## Supporting information

S1 File Consort checklist. (DOC)

S2 File EPOCH trial protocol. (PDF)
