# Supplementary material for: The impact of a point-of-care testing device on CVD risk assessment completion in New Zealand primary-care practice: A cluster randomised controlled trial and qualitative investigation
Source: PLoS One. 2017 Apr 19;12(4):e0174504. doi: 10.1371/journal.pone.0174504 (PMC5396877; doi:10.1371/journal.pone.0174504)
Supplement: S2 File — (PDF) [file pone.0174504.s002.pdf]

Protocol

EPOCH
(Evaluating a Point-Of-Care device in Heart Healthcare)

The impact of point-of-care testing on cardiovascular risk assessment completion in primary care practice

(Trial Registration Number ACTRN12613000607774)

Principal Investigators

Dr Sue Wells
Dr Natasha Rafter
National Institute for Health Innovation, The University of Auckland
09 373 7999

National Institute for Health Innovation

© The University of Auckland, 2013

EPOCH Study Co-ordinating Centre

Postal address
National Institute for Health Innovation
School of Population Health
The University of Auckland
Private Bag 92019
Auckland Mail Centre
Auckland 1142
New Zealand
Tel: 64 9 373 7599 ext 84765
Fax: 64 9 373 1710
Email: epoch@nihi.auckland.ac.nz

Street address
National Institute for Health Innovation
Level 4, School of Population Health
Tamaki Campus
The University of Auckland
261 Morrin Road
Glen Innes
Auckland 1072
New Zealand

© The University of Auckland, 2013

Advisory Group Members

**A/Professor Susan Wells**<br>Co-Principal Investigator	Epidemiology and Biostatistics<br>School of Population Health<br>University of Auckland<br>Private Bag 92019<br>Auckland 1142, New Zealand<br>Tel: 09 373 7599 ext. 82463<br>Fax: 09 373 1710<br>Emergency no: 021 442 558<br>Email: s.wells@auckland.ac.nz
**Dr Natasha Rafter**<br>Co-Principal Investigator	Population Health Sciences<br>Royal College of Surgeons in Ireland<br>123 St Stephens Green<br>Dublin 2<br>Ireland<br>Tel: +353 89 411 0605<br>Email: natasharafter@rcsi.ie
**Dr Kyle Eggleton**<br>Clinical Director Primary Healthcare	Manaia Health PHO<br>PO Box 1878<br>Whangarei 0140<br>Tel:09 438 1015<br>Fax: 09 438 3210<br>Email: kyle@manaiapho.co.nz
**Rose Lightfoot**<br>Chief Executive Officer	Te Tai Tokerau PHO<br>PO Box 507<br>Kaitaia 0441<br>Tel: 09 408 3142<br>Fax: 09 408 3152<br>Email: rose@tttpho.co.nz
**Kim Arcus**<br>Manager Heart Healthcare	Heart Foundation<br>National Office<br>9 Kalmia St<br>Auckland 1051<br>PO Box 17-160 Auckland 1546<br>Tel: 09 571 9185<br>Email: KimA@heartfoundation.org.nz
**Professor Chris Bullen**<br>Director	National Institute for Health Innovation<br>University of Auckland<br>Private Bag 92019<br>Auckland 1142, New Zealand<br>Tel: 09 373 923 4730<br>Fax: 09 373 1710<br>Email: c.bullen@nihi.auckland.ac.nz

© The University of Auckland, 2013

**Geoff Herd**<br>**Point of care testing coordinator**	Northland District Health Board<br>Whangarei Hospital<br>2 Hospital Road<br>Private Bag 9742<br>Whangarei 0148<br>Tel: 09 430 4100<br>Email: Geoff.herd@northlanddhb.org.nz
**A/Prof Tim Kenealy**<br>Associate Professor of Integrated Care	South Auckland Clinical School<br>University of Auckland<br>Middlemore Hospital<br>Private Bag 93311<br>Auckland 1640<br>Tel:64 274 905 914<br>Email: t.kenealy@auckland.ac.nz
**Dr Tania Riddell**<br>Research Fellow	Epidemiology and Biostatistics<br>School of Population Health<br>University of Auckland<br>Private Bag 92019<br>Auckland 1142, New Zealand<br>Tel: 09 373 7599 ext. 86333<br>Email: t.riddell@auckland.ac.nz

Study Management Committee Members

**Dr Natasha Rafter**<br>Co-Principal Investigator	Population Health Sciences<br>Royal College of Surgeons in Ireland<br>123 St Stephens Green<br>Dublin 2<br>Ireland<br>Tel: +353 89 411 0605<br>Email: natasharafter@rcsi.ie
**A/Professor Susan Wells**<br>Co-Principal Investigator	Epidemiology and Biostatistics<br>School of Population Health<br>University of Auckland<br>Private Bag 92019<br>Auckland 1142, New Zealand<br>Tel: 09 373 7599 ext. 82463<br>Fax: 09 373 1710<br>Emergency no: 021 442 558<br>Email: s.wells@auckland.ac.nz
**Angela Wadham**<br>Project Manager	National Institute for Health Innovation<br>University of Auckland<br>Private Bag 92019<br>Auckland 1142, New Zealand<br>Tel: 64 9 923 4662<br>Fax: 64 9 373 1710<br>Emergency no: 021406463<br>Email: a.wadham@auckland.ac.nz

© The University of Auckland, 2013

**Varsha Parag**<br>Senior Biostatistician	National Institute for Health Innovation<br>University of Auckland<br>Private Bag 92019<br>Auckland 1142, New Zealand<br>Tel: 64 9 923 4710<br>Fax: 64 9 373 1710<br>Email: v.parag@nihi.auckland.ac.nz
**John Fa'atui**<br>Data Manager	National Institute for Health Innovation<br>University of Auckland<br>Private Bag 92019<br>Auckland 1142, New Zealand<br>Tel: 64 9 373 7599 ext. 84552<br>Fax: 64 9 373 1710<br>Email: j.faatui@nihi.auckland.ac.nz
**Colleen Ng**<br>Developer	National Institute for Health Innovation<br>University of Auckland<br>Private Bag 92019<br>Auckland 1142, New Zealand<br>Tel: 64 9 373 7599 ext. 84737<br>Fax: 64 9 373 1710<br>Email: c.ng@nihi.auckland.ac.nz
**Professor Chris Bullen**<br>Director	National Institute for Health Innovation<br>University of Auckland<br>Private Bag 92019<br>Auckland 1142, New Zealand<br>Tel: 09 373 923 4730<br>Fax: 09 373 1710<br>Email: c.bullen@nihi.auckland.ac.nz

Study Centres

Te Tai Tokerau PHO	PO Box 507<br>Kaitaia<br>Tel: 64 9 408 3142
Manaia Health PHO	PO Box 1878<br>Whangarei<br>Tel: 64 9 438 1015

Project Sponsors

Roche Diagnostics New Zealand Ltd
15 Rakino Way
Mt. Wellington
Auckland 1060
New Zealand
Tel: 09 276 4157
Fax: 09 276 8917

Source of Study Treatment

Roche Diagnostics International Ltd<br>Roche Diagnostics NZ Ltd	Letitia O'Dwyer<br>Medical Marketing Manager<br>Roche Diagnostics NZ Ltd<br>15 Rakino Way<br>Mt. Wellington<br>Auckland 1060<br>New Zealand<br>Tel: 09 276 4157<br>Fax: 09 276 8917<br>Email: Letitia.odwyer@roche.com

Material name (Product name)	Material Number
Cobas b101	06378668190
HbA1c disc	06378676190
HbA1c control	06380204190
Lipid panel disc	06380115190
Lipid panel control	06380182190
5 x Safe-T-Pro Plus Lancing device	03603539200

© The University of Auckland, 2013

Signature Page

Author(s):

**Authors Name**	**Signature:**	**Date:**
A/Prof Sue Wells		29/4/2015

**NIHI Signatory: Director**	**Signature:**	**Date:**
Professor Chris Bullen		29/4/2015

**Revision Chronology:**	Date	Type *e.g. Original*
Version 1	29 May 2013	Original
Version 2	31 July 2013	Amendment 1
Version 3	5 August 2014	Amendment 2
Version 4	20 April 2015	Amendment 3

© The University of Auckland, 2013

Table of contents

Contents

EPOCH STUDY CO-ORDINATING CENTRE 2
ADVISORY GROUP MEMBERS 3
STUDY MANAGEMENT COMMITTEE MEMBERS 4
STUDY CENTRES 5
PROJECT SPONSORS 5
SOURCE OF STUDY TREATMENT 6
SIGNATURE PAGE 7
TABLE OF CONTENTS 8
1. OVERVIEW 10
2. STUDY PLAN SCHEMATIC 12
3. BACKGROUND 13
3.1 CVD RISK ASSESSMENT 13
3.2 PREDICT CVD SOFTWARE TOOL 14
3.3 ASSESSMENT OF CHOLESTEROL AND DIABETES 14
3.4 POINT-OF-CARE TESTING FOR CHOLESTEROL AND HBA1C 14
4. RATIONALE FOR THE PRESENT STUDY 16
5. STUDY OBJECTIVES 17
6. STUDY DESIGN 18
6.1 INCLUSION CRITERIA 18
6.2 EXCLUSION CRITERIA 19
6.3 RECRUITMENT 20
6.4 STUDY GROUPS 20
6.5 RANDOMISATION 20
6.6 BLINDING 20
6.7 WITHDRAWAL CRITERIA 21
6.8 PRIMARY OUTCOME MEASURE 21
6.9 SECONDARY OUTCOME MEASURES 21
6.10 TRAINING 21
6.11 PRACTICE FOLLOW-UP 21
6.12 DETAIL OF DATA COLLECTION 21
6.12.1 Eligibility, randomisation and baseline assessment 21
6.12.2 CVD risk assessment data collection 21
6.12.3 Qualitative assessment of practice acceptability 22
7. STATISTICAL CONSIDERATIONS 24
7.1 SAMPLE SIZE 24
7.2 STATISTICAL ANALYSES 24
7.2.1 Baseline characteristics 25
7.2.2 Study effects 25
7.2.3 Run charts 25
7.2.4 Cost analysis 25
7.2.5 Procedures to account for missing data 25
7.2.6 Interim analyses 26
7.3 DATA MANAGEMENT 26

© The University of Auckland, 2013

7.3.1 PREDICT data security- submission, access and storage ... 26

8. ETHICAL APPROVAL ... 28

9. ASSESSMENT OF SAFETY / ADVERSE EVENT REPORTING ... 29

10. COBAS B 101 DEVICE SUPPLIES ... 30

10.1 PRODUCT IDENTIFICATION ... 30
10.2 HANDLING OF COBAS B 101 PRODUCTS ... 30
10.3 SUPPLY RECORDS ... 30
10.4 STUDY MEDICAL DEVICE ON COMPLETION OF STUDY ... 30

11. RELEVANCE TO HEALTH ... 31

12. DISSEMINATION OF RESULTS ... 32

12.1 STUDY REGISTRATION ... 32
12.2 STUDY PRACTICES ... 32
12.3 THE GENERAL PUBLIC ... 32
12.4 ACADEMIC/PROFESSIONAL COLLEAGUES ... 32
12.5 HEALTH SERVICE FUNDERS AND PROVIDERS ... 32
12.6 IWI/ MĀORI ... 32

13. ADMINISTRATIVE SECTION ... 33

13.1 ADHERENCE TO THE PROTOCOL ... 33
13.2 PROTOCOL REVISION PROCEDURES ... 33
13.3 CASE REPORT FORM PROCEDURES ... 33
13.4 MONITORING/ SOURCE DOCUMENT VERIFICATION ... 33
13.5 DATA CONFIDENTIALITY AND SECURITY ... 33
13.6 REPORTING SCHEDULE ... 33
13.7 RECORD RETENTION POLICY ... 33
13.8 INSURANCE ... 33
13.9 OWNERSHIP OF DATA AND PUBLICATION POLICY ... 34

14. REFERENCES ... 35

15. STUDY ACKNOWLEDGEMENT ... 36

16. APPENDIX 1 – TERMS OF REFERENCE ... 37

16.1 ADVISORY GROUP ... 37
16.2 STUDY MANAGEMENT COMMITTEE ... 37

17. APPENDIX 2 – STUDY INFORMATION SHEET ... 38

18. APPENDIX 3 – COBAS B 101 MEDICAL DEVICE ... 40

19. APPENDIX 4 - COBAS B 101 QUALITY ASSURANCE ... 41

20. APPENDIX 5 – PROPOSED TIMELINE ... 43

21. APPENDIX 6 – SUMMARY OF PROTOCOL AMENDMENTS ... 44

© The University of Auckland, 2013

1. Overview

Title of study
EPOCH (Evaluating a Point-Of-Care device in Heart Healthcare)

Investigators and study centres
A/Prof Sue Wells, Dr Natasha Rafter, Prof Chris Bullen, The University of Auckland.
Dr Kyle Eggleton, Manaia PHO.
Rose Lightfoot, Te Tai Tokerau PHO.

Study period
Expected duration 3-6 months (dependent on practices' rate of risk assessment).

Objectives
To evaluate the impact of POC testing for lipids (TC/HDL) and diabetes (HbA1c) on the frequency of completed cardiovascular disease (CVD) risk assessments in the eligible population in primary care practice. The primary aim of this study is to determine whether POC testing for lipids (TC/HDL) and diabetes (HbA1c) is at least as effective as the control (laboratory) on the frequency of completed cardiovascular disease (CVD) risk assessments in the eligible population at 12 months in primary care practice.

Study design and methodology
Pragmatic, prospective, open, blinded endpoint, cluster randomised controlled trial with non-inferiority design. Twenty general practices: 10 randomised to receive the POC device, 10 continue with laboratory testing for lipids and diabetes.

Study population
Patients aged 35-79 years who are eligible for CVD risk assessment.
Number of subjects
Minimum of 1000 risk assessments (approximately 50 per practice).

Main criteria for inclusion
Adults aged 35-79 years who are eligible for CVD risk assessment based on age, sex, ethnicity, previous CVD risk assessment).

Exclusion criteria
Ineligible for CVD risk assessment, or have a previous completed CVD risk assessment within the recommended time. Aged < 35 years or ≥ 80 years.

POC group
Cobas b 101: finger prick point-of-care testing medical device for total cholesterol/ high-density lipoprotein (TC/HDL) and haemoglobin type A1c (HbA1c).

Laboratory (control) group
Usual laboratory testing for TC/HDL and HbA1c.

Criteria for evaluation
Primary outcome
Completed CVD risk assessments at end of study.

Secondary outcomes
Incomplete ("parked") CVD risk assessments at end of study.

© The University of Auckland, 2013

Completed CVD risk assessment by age, sex, Māori/non Māori, diabetes, smoking status, deprivation quintile, practice location (urban/rural), practice size, previous CVD risk score.
Average duration of CVD risk assessment.
Cost analysis.
Acceptability, feasibility and practice impact of POC testing for TC/HDL and HbA1c in general practice.

Statistical methods

Study power

Assuming an intraclass correlation coefficient of 0.025, a two sided 5% significance, a sample size of 10 practices with an average of 50 patients each, in each of the two arms, will confer 90% power to detect a difference in completed CVD risk assessments of 10-15% between the POC practices and laboratory practices.

Statistical analysis

Intention-to-treat analysis of the primary outcome. Generalized estimating equations (GEE) or random effects mixed models will be used. Statistical process control charts (run charts) will demonstrate monthly CVD risk assessment.

Funding

Roche Diagnostics International Limited.
Roche Diagnostics NZ Limited.

© The University of Auckland, 2013

2. Study Plan Schematic

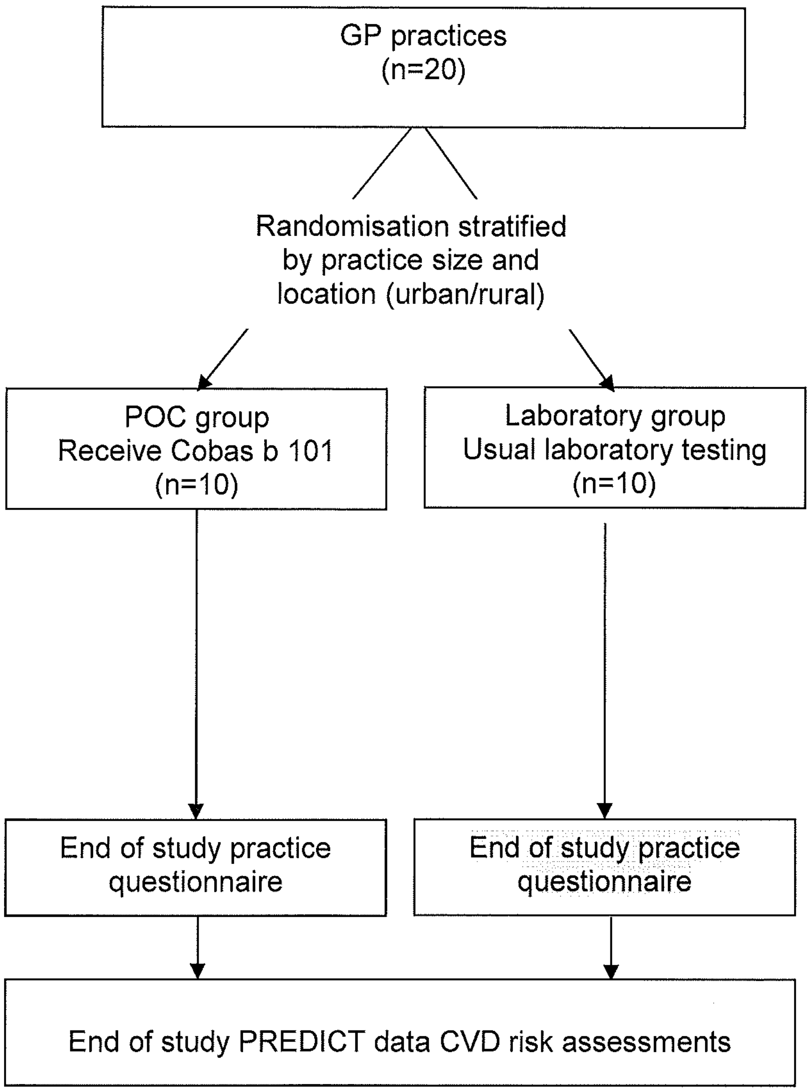

Abbreviations: GP = general practitioner, CVD = cardiovascular disease

© The University of Auckland, 2013

3. Background

3.1 CVD risk assessment

Cardiovascular diseases (CVD) are readily preventable long term conditions that contribute to major ethnic and socioeconomic disparities in New Zealand. Interventions are available that could halve cardiovascular events (such as myocardial infarction and stroke) but the magnitude of treatment benefit is directly proportional to the magnitude of pre-treatment absolute CVD risk.

New Zealand CVD management guidelines[1] recommend identifying people at high risk of developing CVD using a modified version of the Framingham risk prediction equation. Screening is recommended for all men over 45 years, all women over 55 years and 10 years earlier for people who are of Māori (indigenous New Zealanders), Pacific or South Asian ethnic groups, or people with known CVD risk factors. The intensity of management is then tailored according to a patient's five-year CVD risk.

Recently, new CVD and diabetes health targets for healthcare organizations have been introduced by the New Zealand Ministry of Health (MoH).[a] From January 2012 the Ministry will measure the number of completed CVD risk assessments for all eligible persons within the last five years (including diabetes checks). Data will be sourced from the Primary Health Organisation (PHO) performance programme. To assist District Health Boards (DHBs) in achieving the target of 90%, interim targets have been set over the next two years. The first is 60% by 1 July 2012, then 75% by 1 July 2013, and full achievement of the national target of 90% by 1 July 2014. PHOs and their member general practitioners (GPs) are well-informed of these targets via a number of mechanisms. These include PHO performance being annually benchmarked against others, PHOs receiving incentive payments (albeit modest) according to CVD risk assessment performance and planned PHO activities encouraging practice compliance such as CME/CNE and other practice interventions (such as visiting facilitators).

Up to 2011, we estimated that only about one third of the eligible population from the combined Northland and Auckland regions had been risk assessed[2] and this was mainly via opportunistic screening.[2] However, in the last 18 months there has been a marked acceleration of CVD risk assessment activity with some PHOs reporting 60%-70% screening of their eligible population.

The ultimate aim of the MoH is to shift the measure of quality from the proportion who have had a CVD risk assessment to the proportion who have received effective management (e.g. prescription uptake and medication adherence). Targets incorporating effective management are likely to be developed post 2014. Recommended CVD risk management is currently not being systematically offered to patients based on their level of CVD risk. New Zealand studies indicate that both over- and under-use of cardiovascular interventions is common in both primary and secondary prevention.[3, 4] A recent audit of patients with established CVD found that while most were receiving aspirin, 30% were not taking a statin and a further 30% were not prescribed any blood pressure (BP) lowering drug.[3] An audit of patients without a history of CVD visiting their general practitioners in 2000 showed that there was little evidence that CVD risk management was being targeted to those at highest risk.[4]

---

[a] http://www.health.govt.nz/new-zealand-health-system/health-targets/2011-12-health-targets/health-targets-better-diabetes-and-cardiovascular-services-more-heart-and-diabetes-checks

© The University of Auckland, 2013

3.2 PREDICT CVD software tool

All GPs in Northland have access to the PREDICT CVD risk assessment and management tool. PREDICT is a web-based decision support system used since 2002 in New Zealand in a variety of settings - general practices, community outreach screening, workplaces (occupational health assessments) and in secondary care (Coronary Care Units and Medical Outpatient Clinics). Integrated with primary care patient management systems (PMS), PREDICT provides clinicians and patients with tailored evidence-based CVD risk prediction and management support. As of the last quarter of 2012, 360,000 New Zealanders in primary and secondary care have had a PREDICT CVD assessment. In addition, risk communication software, Your Heart Forecast, is integrated with the PREDICT platform.[5] PREDICT and associated research has been supported through Health Research Council and National Heart Foundation funding.

The PREDICT CVD risk assessment and management program has been fully integrated with the electronic patient medical record that is used at the time of consultation. When a primary care provider wishes to do a CVD risk assessment they simply open the CVD risk assessment window. Items such as the New Zealand National Health Index (NHI number) unique patient identifier, ethnicity, laboratory results (if available), blood pressure recordings, prior history of CVD or diabetes, will be automatically populated from the electronic patient record into the appropriate fields of the template.

When a clinician uses PREDICT for CVD risk assessment and/or management, they submit patient data to the PREDICT server and within seconds receive a CVD risk calculation for the patient as well as guideline recommendations tailored to an individual's CVD risk profile. A copy of this transaction is saved both on the electronic patient medical record and in the PREDICT server. Once a risk assessment is conducted the provider can go directly to the Heart Forecast via PREDICT to begin discussing with the patient what their CVD risk is, their burden of modifiable disease and suggest appropriate evidence-based management.

3.3 Assessment of cholesterol and diabetes

As part of a CVD risk assessment, the New Zealand guidelines recommend blood lipid testing and use of the ratio of total cholesterol to high density lipoprotein (TC/HDL, fasting or non-fasting) in the risk calculation. In 2009, screening for diabetes was also recommended as part of the risk assessment using either fasting glucose or haemoglobin type A1c (HbA1c, fasting or non-fasting).

Currently, if the lipid and diabetes values are not known at the visit, the GP or practice nurse must order a blood test (via a laboratory referral form). Transmission from the screen to the PREDICT server will not occur unless all the data fields are filled in. If screening blood tests are not available the assessment can be "parked" and saved until all data is subsequently available. Ideally then a risk assessment would be completed with the patient on a second visit. It is not known what proportion of risk assessments remain incomplete due to the laboratory testing step and necessity for two consultations.

3.4 Point-of-care testing for cholesterol and HbA1c

A new point-of-care (POC) device (Cobas b 101 developed by Roche Diagnostics) that can perform both TC/HDL and HbA1c tests has recently become available on the New Zealand and global markets. It is estimated that a thirty minute consultation could provide a completed CVD risk assessment (see figure below and Appendix 3). This includes 15 minutes to undertake the finger prick sampling and provide the test results. The Cobas b 101

practitioner to complete the risk assessment and provide tailored advice in the one consultation.

© The University of Auckland, 2013

CVD Risk Assessment

cobas b 101 local study proposal

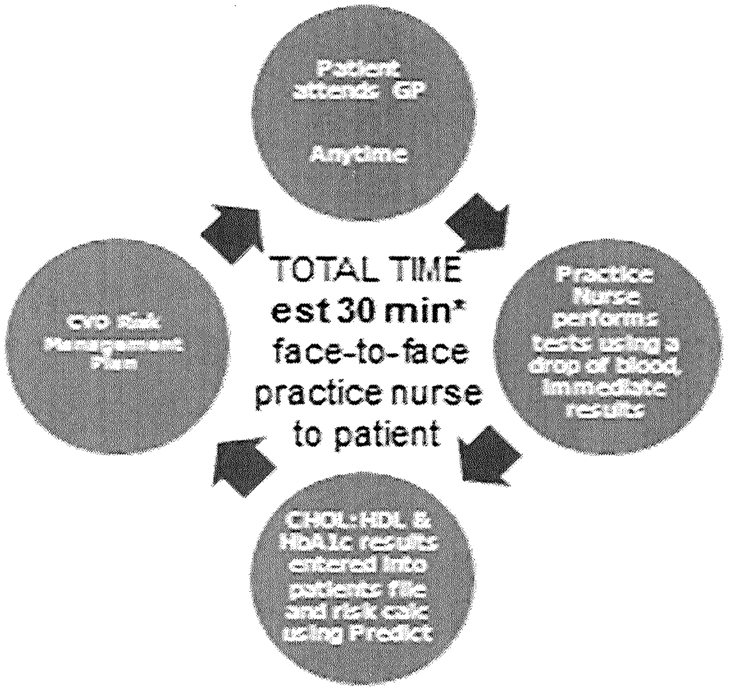

cobas b 101 – The HbA1c & Lipid Monitoring System

Two Tests from One Finger Prick – Possible Through Heparin Coating

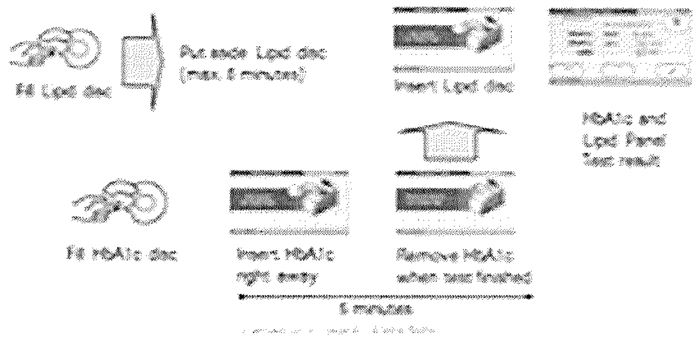

cobas®

4. Rationale for the Present Study

Anecdotally many people given blood test forms do not subsequently go to a community laboratory to have the blood taken for lipids and diabetes testing. Without these test results full CVD risk assessment is not possible, and recommended care cannot be undertaken. The uptake of such screening tests has been reported as being more of a problem in rural areas and for those who are most socio-economically deprived.[6-9]

While the Prompt evaluation study[10] indicated no disparities in the GP CVD risk assessment process between Māori and non Māori, a recent study (Moss et al. 2013 unpublished) has found that cholesterol testing among Māori with established CVD living in the Auckland region is lower than European and lower than that found for other high risk ethnic groups such as Pacific and Indian peoples.

Point of care (POC) testing provides biological sample results rapidly at the time of consultation, and thus overcomes this barrier to care. In the context of CVD risk assessment, it would enable GPs to move seamlessly in one patient consultation from completed CVD risk assessment using PREDICT, to CVD risk communication and commencing or altering CVD preventive management.

The POC device Cobas b 101 can perform both TC/HDL and HbA1c tests and therefore provide results immediately to practitioner and patient. However, its use in primary care settings has not been evaluated. In particular, it is important to determine whether access to such POC testing for TC/HDL and HbA1c could increase the proportion of the eligible population with completed CVD risk assessments. Furthermore, it is important to evaluate whether the introduction of new technologies will not increase disparities in screening particularly for high risk ethnic groups and those living in rural or deprived communities.

This is a unique opportunity to improve care processes for patients, build on existing primary care relationships, integrate POC testing with existing health information technologies, and evaluate how POC testing impacts CVD risk assessment. Furthermore this proposed research will:

- be the first study to document the costs of POC testing for CVD risk assessment compared with laboratory testing in New Zealand primary care, and
- qualitatively evaluate the acceptability, feasibility and practice impact of POC testing in routine New Zealand primary care.

© The University of Auckland, 2013

5. Study Objectives

Primary study objective:

To evaluate the impact of POC testing for lipids (TC/HDL) and diabetes (HbA1c) on the frequency of completed CVD risk assessments in the eligible population. The primary aim of this study is to determine whether POC testing for lipids (TC/HDL) and diabetes (HbA1c) is at least as effective as the control (laboratory) on the frequency of completed cardiovascular disease (CVD) risk assessments in the eligible population at 12 months in primary care practice.

Secondary study objective(s):

1. To evaluate the impact of POC testing for TC/HDL and HbA1c on the frequency of incomplete ("parked") CVD risk assessments in the eligible population.
2. To evaluate the impact of POC testing for TC/HDL and HbA1c on CVD risk assessments in the eligible population by age, sex, deprivation quintile, for Māori and non Māori, diabetes, smoking status, and previous CVD risk score.
3. To evaluate the impact of POC testing for TC/HDL and HbA1c on the average duration of CVD risk assessment.
4. To evaluate whether the impact of POC testing is the same in urban areas (e.g. Whangarei City) as in rural areas and in large and small GP practices.
5. To conduct an cost analysis of POC testing compared with laboratory testing.
6. To evaluate the acceptability, feasibility and practice impact of POC testing for TC/HDL and HbA1c in general practice.

© The University of Auckland, 2013

6. Study Design

This is a pragmatic, prospective, open, blinded endpoint, cluster randomised controlled non-inferiority trial in primary care.

The study setting is the patients enrolled in GP practices within the Northland region of New Zealand that belong to either Manaia Health Primary Healthcare Organization (PHO) or Te Tai Tokerau PHO. Manaia Health has 24 practices and Te Tai Tokerau has 15. A cluster randomised controlled trial is proposed within twenty of these general practices who use PREDICT and agree to participate in the study.

The eligible population is comprised of patients enrolled in these practices who are aged between 35-79 years, who as determined by their GP, meet age, sex and ethnicity guideline criteria for CVD risk assessment (Table1) and do not have a previous recorded CVD risk assessment or if they have had a previous CVD risk assessment are overdue for their next check (Table 2). All CVD risk assessment data will be automatically captured within the PREDICT server before, during, and after the study as part of usual clinical practice.

The unit of randomisation will be the practice and data collection will be at the individual patient level. The study involves random allocation of the practices to either the POC group (Cobas b 101) or laboratory (control) group. A cluster RCT design was necessary because the cobas b 101 device will be provided to the practice as a cluster level intervention.

6.1 Inclusion criteria

Patients will be included in the eligible study population if

- they are aged at least 35 years and less than 80 years old, and
- as determined by their GP, they meet national age, sex and ethnicity guideline criteria for CVD risk assessment (Table 1) and repeat assessment recommendations (Table 2)

© The University of Auckland, 2013

Table 1 Age to start cardiovascular disease and diabetes risk assessment

Table 1 The age to start cardiovascular disease and diabetes risk assessment

Group	Men	Women
Asymptomatic people without known risk factors	Age 45 years	Age 55 years
Māori, Pacific peoples or people from the Indian subcontinent*	Age 35 years	Age 45 years
People with other known cardiovascular risk factors or at high risk of developing diabetes<br>**Family history risk factors**<br>• Diabetes in first-degree relative (parent, brother or sister)<br>• Premature coronary heart disease or ischaemic stroke in a first-degree relative (father or brother <55 years, mother or sister <65 years)<br>**Personal history risk factors**<br>• People who smoke (or who have quit only in the last 12 months)<br>• Gestational diabetes, polycystic ovary syndrome<br>• Prior blood pressure (BP) ≥160/95 mm Hg, prior TC:HDL ratio ≥7<br>• Known IGT (impaired glucose tolerance) or IFG (impaired fasting glucose)<br>• BMI ≥30 or truncal obesity (waist circumference ≥100 cm in men or ≥90 cm in women)<br>• eGFR† <60 ml/min/1.73 m²	Age 35 years	Age 45 years
People with diabetes	Annually from the time of diagnosis	

* People from the Indian subcontinent = Indian, including Fijian Indian, Sri Lankan, Afghani, Bangladeshi, Nepalese, Pakistani, Tibetan.
† Estimated glomerular filtration rate (eGFR).

Table 2 Frequency of CVD and diabetes risk assessment

Table 4 Frequency of cardiovascular risk assessment

5-year risk <5%	Further risk assessment in 10 years
5-year risk 5–10%	Further risk assessment in 5 years
5-year risk 10–15%	Further risk assessment in 2 years
5-year risk ≥15%, diabetes, or on lipid or BP lowering medication	Annual risk assessment

6.2 Exclusion criteria

Patients will be excluded if they one or more of the following:

- are under 35 years old
- are 80 years old or older
- are not eligible for CVD risk assessment.

© The University of Auckland, 2013

6.3 Recruitment

The PHOs will invite practices to join the study. Interested practices will be visited and twenty practices who agree to be part of the study will be randomised. The eligible population will be identified automatically and anonymously through an existing CVD population register held by the PHOs and PREDICT. Notices informing patients about the study will be placed in GP waiting rooms of practices who have been allocated to the POC group and an Information Sheet will be available (Appendix 2).

6.4 Study groups

- **POC group:**

The 10 practices randomised to the POC group will receive the following materials and training in their use from Roche Diagnostics.

Table 3 Cobas b 101 device and consumables

Material name (Product name)	Material Number	Unit
Cobas b101	06378668190	each
HbA1c disc	06378676190	Box of 10
HbA1c control	06380204190	2 vials
Lipid panel disc	06380115190	Box of 10
Lipid panel control	06380182190	2 vials
5 x Safe-T-Pro Plus Lancing device	03603539200	Box of 200

- **Laboratory group:**

The 10 practices randomized to the laboratory group will continue current practice of requesting laboratory testing to complete CVD risk assessment.

6.5 Randomisation

Participating practices will be randomised by the biostatistician into POC and laboratory groups. Randomisation will be stratified by practice size (number of effective FTE GPs where 0.8 FTE = 1.0 effective FTE: <2 effective FTE small, ≥2 effective FTE large)) and rurality of location (urban [within the Whangarei city boundary] or rural). As these factors are potentially associated, directly or indirectly, with the probability of CVD risk assessment completion; stratification will ensure a balance in these potential confounders between the two study arms.

6.6 Blinding

This is a single blind trial. Whilst it is not possible to blind practices to having the Cobas b 101 or not, study academic investigators will analyse the CVD risk assessment endpoint data blinded to the practice allocation to POC or laboratory group. This data will be collected through the PREDICT CVD risk assessment programme as part of routine clinical practice. Study staff will have no influence or involvement in patient clinical data collection or recording. The PREDICT data will be provided to the investigators by practice separated into two groups. At the end of the analysis the allocation assignment to POC or laboratory group will be revealed to the investigators.

© The University of Auckland, 2013

6.7 Withdrawal criteria

Patients will receive CVD risk assessment as part of normal clinical care which includes testing for lipids and diabetes. For patients in the POC group, a laboratory test may be requested by the health care practitioner or participant, instead of, or in addition to, point-of-care testing. This will not withdraw the patient from the study.

6.8 Primary outcome measure

- Completed CVD risk assessment at end of study.

6.9 Secondary outcome measures

- Incomplete ("parked") CVD risk assessment at end of study.
- Completed CVD risk assessment by age, gender, Māori/non Māori, diabetes, smoking status, deprivation quintile, practice location (urban/rural), practice size, , previous CVD risk score.
- Average duration of CVD risk assessment.
- Cost analysis.
- Acceptability, feasibility and practice impact of POC testing for CVD risk assessment in general practice.

6.10 Training

Roche Diagnostics will be responsible for the training of practice staff in the use of the device and ongoing quality requirements (e.g. monthly control tests). A quality assurance programme will be run alongside the training and will include some parallel testing of training samples to ensure that staff who are being trained are achieving optimum results on the cobas 101 when compared with conventional laboratory testing

6.11 Practice follow-up

Each of the ten practices allocated to the POC group will undergo a training session in the use of the Cobas b 101 device. About half way through the study e.g. at 3 months, and at the end of the study practices will be contacted by PHO facilitators to document any additional staff time and/or other resources required by the use of the Cobas b 101 device and to assess its overall acceptability, feasibility and practice impact. The study will be continued until at least 1000 risk assessment have been performed and all the point of care consumables have been utilised by the POC group. It is estimated therefore the study duration will be in the order of 3-6 months.

6.12 Detail of data collection

6.12.1 Eligibility, randomisation and baseline assessment

All practices in Manaia and Te Tai Tokerau PHOs will be eligible to be included in the study. The first twenty to provide informed consent will be randomised. A de-identified list of eligible patients in the participating practices will be generated using the Northland PHOs' population register provided by PREDICT.

6.12.2 CVD risk assessment data collection

CVD risk assessment data will be collected by the PREDICT software. At the end of the study the investigators will receive de-identified PREDICT data by practice on the study population who have had a completed or parked CVD risk assessment. The PREDICT data will be provided to the investigators by practice separated into two groups. At the end of the analysis the allocation assignment to POC or laboratory group will be revealed to the investigators.

© The University of Auckland, 2013

6.12.3 Qualitative assessment of practice acceptability

The qualitative assessment (practice questionnaire) will collect information from practices allocated to the POC group on where the POC testing was done, who did it, how user friendly was the tool, how user friendly was the integration with the PREDICT CVD risk assessment, what extra time and resources were required, what changes in work flow were required, any teething difficulties or issues arising during the time period, and potential opportunity costs imposed by having the Cobas b 101 device in the practice.

A short questionnaire will also be administered to the control practices asking about current practice for obtaining HbA1c and TC/HDL blood results, the major barriers to CVD risk assessment in their practice and their perceptions about the acceptability and feasibility of having the cobas b 101 device in the practice.

© The University of Auckland, 2013

Table 1: Details of follow-up

**Timing**<br><br>**Description**<br><br>**Form**	**Week 0**<br><br>**Practice randomisation**	**Week 0**<br><br>**PREDICT**	**End of study**<br><br>**Practice F/U**<br><br>**Questionnaire**	**End of study**<br><br>**PREDICT**
Randomisation	X			
Study eligible population		X		
Useability questionnaire			X	
CVD risk assessments				X
Laboratory and Cobas b 101 data				X

© The University of Auckland, 2013

7. Statistical Considerations

7.1 Sample size

The study will involve a two-arm parallel cluster randomised controlled trial (CRCT) design with 20 practices (10 practices per arm). The primary outcome is whether the eligible patients have had a completed CVD risk assessment during the study. Preliminary analyses indicated that at least 70% of the enrolled populations within Manaia and Te Tai Tokerau PHOs have had a completed CVD risk assessment since 2006. Many of these people will be due for repeat CVD risk assessment. Significant delays to the conduct of this trial have occurred both prior to commencement and having to halt the trial due to manufacturing problems of the HbA1c discs. During this time practices have accelerated their CVD risk assessments in order to meet the national target (by July 2014 that 90% of the eligible population have had a CVD risk assessment in the previous 5 years.) As at April 2014, the two PHOs have screened approximately 80% of their eligible enrolled populations over the last five years. Therefore this trial now needs to adopt **a priori a non-inferiority hypothesis**.

Our previous sample size calculations May 2013, were as follows; We assumed a primary event rate of 70% in the laboratory group and a 10-15% increase to 85% in the POC group. This figure is based on a study set in community pharmacies where POC testing for cholesterol risk management led to a 10% increase.[11] Taking into account an intraclass correlation coefficient of 0.025 as suggested by Elley et al (2005) from three New Zealand CRCTs[12]; a 5% two-sided level of significant test and study power of 90%, we estimate a minimum sample size of 1000 risk assessments is needed (on average 50 patients per practice).

Our updated sample size calculations are based on analyses estimating the number of eligible people who are eligible for first risk assessment and those that will require a follow-up CVD risk assessment (for example those over 15% CVD risk, and people with diabetes are recommended for annual screening). Within a 12 month period (Aug 2012-2013) it appeared that about 30% of the eligible population had a CVD risk assessment. We assume a primary event rate of 30% in the laboratory group and a 10% increase to 40% in the POC group. This figure is based on a study set in community pharmacies where POC testing for cholesterol risk management led to a 10% increase.[11] The non-inferiority margin is set at 5%. Taking into account an intraclass correlation coefficient of 0.025 as suggested by Elley et al (2005) from three New Zealand CRCTs[12]; a 0.025 one-sided level of significance and study power of 90%, we estimate a minimum sample size of 940 risk assessments (470 in each group) and 10 practices per group is needed assuming an average of 50 patients per practice. Therefore, the sample size is similar to the previous power calculations.

7.2 Statistical analyses

This study has been designed with the assistance of statisticians at the NIHI. These statisticians will also advise and assist with the analysis of the study results. Individual patient's data entered into the PREDICT decision support programme will be imported into an Oracle database at the NIHI using the encrypted NHI, and then extracted into SAS (version 9.3) for analysis. Data analyses will be carried out on an intention-to-treat basis, and will be specified a priori a statistical analysis plan prepared by the study statistician (and agreed upon by all members of the Advisory Group). No interim analyses will be undertaken. All statistical tests will be two-sided with 5% level of significance.

© The University of Auckland, 2013

7.2.1 *Baseline characteristics*

The following baseline characteristics will be collected: age group, gender, deprivation index quintile, Māori/non Māori ethnicity, previous CVD risk score, and past history of CVD based on previous risk assessment data. Baseline characteristics will be summarised using descriptive statistics. Continuous variables will be described as numbers of observed and missing values, mean, standard deviation, median, minimum and maximum. Categorical variables will be described as frequencies and percentages.

7.2.2 *Study effects*

Generalized estimating equations (GEE) or random effects mixed models will be used in treatment evaluations, adjusting for the effect of clustering within practices and important baseline prognostic factors (e.g. practice location (urban/rural), size (number of GPsAnalysis of the non-inferiority hypothesis for the primary outcome will be evaluated by observing whether the lower limit of a one-sided 97.5% CI (or equivalently a two-sided 95% CI) is above the non-inferiority limit of -5%. In the case that non-inferiority is evident, assessment as to whether POC is superior to the control will be carried out using the same approach but comparing to a zero difference. A per-protocol analysis will also be performed in order to check the robustness of the results. Time series analysis will be conducted to describe changes in patterns of use of the POC device, for example increased use initially followed by a decline to a steady state level. Sensitivity analyses for the rate of CVD risk assessment will then be calculated for the latter period as being more indicative of ongoing community use of the POC device.

Duration of CVD risk assessment will be calculated from the start of the population of the PREDICT CVD risk assessment template to completion, i.e. when the five year CVD risk value is calculated.

7.2.3 *Run charts*

Run charts (or statistical process control [SPC] charts) will be developed to assess the impact of the potential novelty imposed by having the POC testing device, any teething difficulties, changes in work process and the Hawthorne effect from being in a study by tracking risk assessment behaviour in the practices. At the end of the study run charts of monthly CVD risk assessments counts for all of the practices will be generated for the duration of the study and for the 12 months prior. Special cause variation (statistically significant shifts in practice) occurring during that time and their association with PHO or the study will be determined using the standard Shewhart criteria.[12]

7.2.4 *Cost analysis*

A cost analysis will be performed for both groups based on practice consultations and testing for lipids and diabetes (laboratory or POC) from a payer perspective. Data on numbers of practice consultations, laboratory and POC device tests will be sourced from PREDICT. Unit cost data will be obtained from the PHOs, local laboratories and Roche Diagnostics. Qualitative information on other practice resource use will be collected through the practice follow-up. Practice staff costs will be assessed by asking practices about the amount of nurse and GP time required under POC and laboratory testing.

7.2.5 *Procedures to account for missing data*

PREDICT data submitted to the Enigma server is required to be complete in order to get a CVD risk assessment response (CVD risk score and ability to go to Your Heart Forecast). However, it is possible to view incomplete templates. Only those templates where laboratory values are missing (i.e. all other variables are entered) will be counted as "parked" CVD risk assessments.

© The University of Auckland, 2013

7.2.6 *Interim analyses*

None planned.

7.3 Data management

The NIHI servers are located in the University of Auckland's purpose built Tier 3 Data Centre. The servers are protected by several systems including the University of Auckland and NIHI firewalls. The servers are regularly backed up, by both the University of Auckland backup system and NIHI's own backup system. The servers have built in hardware redundancy. The NIHI IT infrastructure platform and network have measures in place to protect privacy; e.g. only authorised personnel have access to data, audit trails, de-identification and aggregation of data before handing over to analysts.

A 10% sample of the PREDICT data will be checked upon importing into the NIHI ORACLE database. Checking will consist of: number of rows inserted into ORACLE match number of rows in the data file and data is correct for each column with the 10% of data being checked.

De-identified data will be stored long term (16 years) by the University of Auckland. Access to anonymous aggregated data will be under the control of the University of Auckland EPOCH research team.

7.3.1 *PREDICT data security- submission, access and storage*

The CVD risk factor and management data submitted from the templates in the GP PMS is sent to PREDICT servers via a secure broadband (HTTPS/SSL) internet connection, the same level of security used for internet banking. This involves AES128–bit encryption which cannot be intercepted between the desktop and server. HTTPS stands for Hyper Text Transfer Protocol (Secure) and SSL for Secure Sockets Layer which describes the transport layer level of security. AES stands for Advanced Encryption Standard which is also known as Rijndael. AES is a block cipher, available as 128- 192- or 256-bit, and has been adopted as an encryption standard by the United States government. AES 192- and 256-bit encryption is used for U.S. classified top secret information. The data is securely stored on remote servers, their location depending on the agreement of the PHOs and other organisations in which PREDICT is used. The storage is managed by Enigma Publishing Ltd on behalf of PHOs and has several layers of security; at user login, database level, data level, network level and physical security level as outlined below.

7.3.1.1 User login security

A requirement requested by the users of the tool was that identifiable patient CVD exposure data submitted to the PREDICT server would always be available by authenticated access at a practice or clinic level to their contributing doctors and nurses. This meant that they could follow an individual patient's history over a series of consultations or audit all their patients who had been risk assessed using PREDICT. Similarly PHOs required a view of risk assessment and management for all patients by clinic within their PHO. To cater for this functionality, Enigma has required user level login security credentials to be given by each user prior to accessing any aspect of PREDICT. This User Account ties the person accessing the system to an organizational unit (e.g. clinic or PHO) that sets access rights to data and user functions accordingly. For the clinic or individual practitioner this can be automated after the first authentic logPage 402 in with the "remember me" function. All user account credentials are stored using AES256bit encryption.

7.3.1.2 Database server security

Each database / product is separately secured using a distinct and complex password. This helps to prevent any potential compromise spreading to unrelated databases or products. The

© The University of Auckland, 2013

database server exists on its' own subnet within the hosting environment and is protected from the outside world and the web hosting segment of the network by a firewall.

7.3.1.3 Data level security

The identifiable data contained within the databases is encrypted before it is written to the database. Should any compromise occur where an unauthorised party was able to access the data directly, the contents of the database would remain unreadable to them having been transformed by AES256bit encryption.

7.3.1.4 Network security

The hosting network is protected from network based attacks at a number of layers. There is a firewall that prevents unauthorised parties from attempting to connect to the network and intrusion detection is implemented within the hosting environment. The network has been designed and segmented in a way which helps to protect from any potential attacks. Remote access to the network is firmly restricted.

7.3.1.5 Physical security

The hosting network is locked in a steel cabinet within a data centre which is alarmed and monitored 24 hours/day.

© The University of Auckland, 2013

8. Ethical Approval

Ethics approval will be sought from the relevant Health and Disability Ethics Committee. Practices will be approached by their PHOs and invited to participate in the study. PHO staff with the help of the researchers will explain the study to potential GP participants. Practice staff will also receive an Information Sheet (Appendix 2). Participation by practices in the study will be entirely voluntary. Those that provide written permission will be entered into the study. One GP partner or the practice manager will sign the permission form on behalf of the practice.

Consent from individual patients will not be sought. The CVD risk assessment takes place as part of usual clinical practice using the PREDICT decision support software. This study provides a marketed POC medical device as an alternative to laboratory testing for use to complete CVD risk assessment. There are no physical risks or side effects for patients from the use of POC testing. The Cobas b 101 device has been validated with international and New Zealand laboratory comparators. No additional visits/admissions or patient time are required from participating patients who will be recruited into this study automatically and anonymously via PHO held CVD registers. GPs and practice nurses will conduct CVD risk assessments as part of routine clinical care. The choice of patient to undergo risk assessment will remain with the clinical staff and will not be directed by the study.

Information will be collected automatically and anonymously by the PREDICT server during the process of risk assessment during routine clinical consultations. NHI identifiers will be encrypted using a one-way encryption process. This de-identified individual patient data will be provided via PREDICT to the study investigators. Notices informing patients about the study will be placed in GP waiting rooms of practices in the POC group and an Information Sheet will be available (Appendix 2).

A waiver of consent will be sought from the Health and Disability Ethics Committee on the basis that there is minimal risk associated with study participation, the study would not be feasible otherwise, and only de-identified data will be available to researchers.[13]

© The University of Auckland, 2013

9. Assessment of Safety / Adverse Event Reporting

Adverse events and serious adverse events will not be collected as part of this study because events related to the skin prick test will be very rare, localised, minor and self-limiting. Likely events include pain at point of testing, possible risk of infection, and skin reactions to any sticking plaster applied to the site after the test has been undertaken.

© The University of Auckland, 2013

10. Cobas b 101 device supplies

10.1 Product identification

Roche Diagnostics International and Roche Diagnostics New Zealand Ltd will supply the following Cobas b 101 devices and consumables to be used in this study by the POC group:

Table 4 Study Cobas b 101 supplies

Material name (Product name)	Material Number	Unit	Quantity
Cobas b101	06378668190	each	10
HbA1c disc	06378676190	Box of 10	198
HbA1c control	06380204190	2 vials	120
Lipid panel disc	06380115190	Box of 10	198
Lipid panel control	06380182190	2 vials	120
5 x Safe-T-Pro Plus Lancing device	03603539200	Box of 200	5

Roche Diagnostics NZ Ltd will supply additional consumables for the training sessions for the POC group and laboratory groups.

10.2 Handling of Cobas b 101 products

Roche Diagnostics NZ Ltd will distribute the Cobas b 101 device and consumables to the POC group at the start of the study.

10.3 Supply records

Supply records of the Cobas b 101 device and consumables will be kept by Roche Diagnostics NZ Ltd. These will be provided to the study investigators on request.

10.4 Study medical device on completion of study

On completion of the study period no further new Cobas b 101 devices or consumables will be supplied to the POC group.

© The University of Auckland, 2013

11. Relevance to Health

New Zealand CVD guidelines recommend cholesterol blood testing (TC/HDL) as part of CVD risk assessment. More recently, CVD screening for diabetes was also recommended using either fasting glucose or HbA1c (fasting or non-fasting). By providing these results in a single consultation a POC testing device has the potential to increase the rate of CVD risk assessment and result in more effective and cost effective management of patients, without increasing existing disparities in CVD risk assessment completion between non-Māori and Māori. However these assumptions need to be evaluated in a clinical trial.

© The University of Auckland, 2013

12. Dissemination of Results

12.1 Study registration

This study is registered with the Australian New Zealand Clinical Trials Registry (www.anzctr.org.au), an organisation that maintains a database of trials in progress to assist with the synthesis of controlled trials.

12.2 Study practices

At the end of the study, all participating practices will receive a letter of thanks for participating in the study, a brief summary of the study results, an outline of their significance and future research plans. A summary of the results for interested patients will also be available via their GPs.

12.3 The general public

The findings will be disseminated via news releases to mass media. In addition, the results will be published on the NIHI, Manaia PHO, and Te Tai Tokerau PHO websites.

12.4 Academic/professional colleagues

The findings will be widely disseminated via reports and publications within relevant clinical newspapers and journals, and conference presentations, e.g. to the RNZCGP, Cardiology and NHF conferences.

12.5 Health service funders and providers

The findings will be widely disseminated via reports and presentations to Manaia and Te Tai Tokerau PHOs, Northland DHB, the MoH and Te Puni Kokiri.

12.6 Iwi / Māori

The findings will be disseminated via news releases to the Māori media and a summary of the study results will be provided to local Māori health providers, Te Puni Kokiri, Te Hotu Manawa Māori, Te ORA (Māori Medical Practitioners Association) and the National Council of Māori Nurses. Results will be presented at appropriate assemblies and hui.

© The University of Auckland, 2013

13. Administrative Section

13.1 Adherence to the protocol

Except for a change that is intended to eliminate an immediate hazard to participants, the approved protocol will be conducted as described. Any significant protocol deviation will be documented in the study files.

13.2 Protocol revision procedures

All revisions will be discussed with, and approved by, the Study Advisory Group. If the revision is an "administrative letter", the principal investigator will submit it to the appropriate Ethics Committee for their information. If the revision is an "amendment", the principal investigator will sign it. The principal investigator will submit the amendment to the appropriate Ethics Committee for review and approval or favourable opinion prior to implementation. Documentation of approval signed by the chairperson or designee of the Ethics Committee will be sent to the principal investigator. This should be filed in the study files and copies where appropriate sent to relevant sites.

If an amendment substantially alters the study design practices currently enrolled in the study, if they are affected by the amendment, will be contacted by telephone and the amendment discussed and verbal consent re-obtained.

13.3 Case report form procedures

The practice questionnaires will be completed by practices allocated to the POC group at the time of the end of study practice visits. Practices will be identified by a study identification number. Information from the questionnaires will be entered into an excel spreadsheet by the researcher.

13.4 Monitoring/ Source document verification

Not applicable.

13.5 Data confidentiality and security

Data will be entered, stored and backed-up in a secure manner via the NIHI internet data management system (See Manual of Procedures).

13.6 Reporting schedule

The Project Manager will provide annual reports of the progress, or completion, termination or discontinuation of the study to the Health and Disability Ethics Committee and Roche Diagnostics NZ Ltd.

13.7 Record retention policy

Essential documents as defined by ICH GCP will be retained by the NIHI (or an approved archiver) for at least 16 years following trial closure (New Zealand Regulatory Guidelines for Medicines 1998).

13.8 Insurance

This research is not conducted principally for the benefit of the manufacturer or distributor of the Cobas b 101 device. Therefore patients may be entitled to compensation from the Accident Compensation Corporation (ACC) for personal injury suffered as a result of use of the device during the study (section 32 (4) of the Injury, Prevention, Rehabilitation and Compensation Act

© The University of Auckland, 2013

2001 and section 13 of the Injury, Prevention, Rehabilitation and Compensation Amendment Act (No 2) 2005).

13.9 Ownership of data and publication policy

All identifiable patient data remain the property of the practices. All data derived through the use of the PREDICT web-based tool for CVD risk assessment and management are held on the PREDICT server on behalf of the contributing PHOs. With permission from PHOs and with formal ethical committee approval, these data have previously been de-identified and extracted for longitudinal quality improvement and risk prediction research (HRC-PREDICT and HRC-VIEW studies).

The EPOCH study will be conducted in a similar way. All CVD risk assessment data derived by the use of the PREDICT decision support tool from consenting and participating practices during the study time period of interest will be extracted. These data will be de-identified at the patient and practice level prior to extraction and delivered securely to NIHI via password protected web transfer functionality. NIHI will then have the responsibility for storage, protection and retrieval of EPOCH study data. The Advisory Group will have the responsibility for the safe guardianship and use of the data. All access, analyses and dissemination of Māori-specific data will be the joint responsibility of the Advisory Group and the NIHI Māori Advisory Committee.

All publications will be approved by members of the Advisory Group, who will be named on all reports. Participating practices, the PHO facilitators, members of the Management Committee who are not part of the Advisory Group, and study sponsors will be acknowledged in the final report and in all publications and presentations resulting from this study.

© The University of Auckland, 2013

14. References

1. New Zealand Guideline Group. The Assessment and Management of Cardiovascular Risk. Wellington, New Zealand; 2003.
2. Wells S. Getting evidence to and from general practice consultations for cardiovascular risk management using computerised decision support. PhD thesis. Auckland: University of Auckland; 2008.
3. El-Jack S, Kerr A. Secondary prevention in coronary artery disease patients in South Auckland: moving targets and the current treatment gap. New Zealand Medical Journal 2003;116:U664.
4. Rafter N, Connor J, Hall J, et al. Cardiovascular medications in primary care: treatment gaps and targeting by absolute risk. New Zealand Medical Journal 2005;118:U1676.
5. Wells S, Kerr A, Eadie S, Wiltshire C, Jackson R. 'Your Heart Forecast'- a new approach for describing and communicating cardiovascular risk? Heart 2010;96:708-13.
6. Dalsgaard EM, Lauritzen T, Christiansen T, Mai KS, Borch-Johnsen K, Sandbaek A. Socioeconomic factors related to attendance at a Type 2 diabetes screening programme. Diabetic Medicine 2009;26:518-25.
7. Fagard RH. The role of exercise in blood pressure control: supportive evidence. Journal of Hypertension 1995;13:1223-7.
8. Franks P, Gold MR, Bell BP, Naumburg EH, Engerman J. Barriers to cholesterol testing in a rural community. Journal of Family Practice 1991;32:614-8.
9. Nijhof N, ter Hoeven CL, de Jong MDT. Determinants of the use of a diabetes risk-screening test. Journal of Community Health 2008;33:313-7.
10. Wells S, Furness S, Rafter N, et al. Integrated electronic decision support increases cardiovascular disease risk assessment four fold in routine primary care practice. European Journal of Cardiovascular Prevention & Rehabilitation 2008;15:173.
11. Tsuyuki RT, Johnson JA, Teo KK, et al. A randomized trial of the effect of community pharmacist intervention on cholesterol risk management: the Study of Cardiovascular Risk Intervention by Pharmacists (SCRIP). Archives of Internal Medicine 2002;162:1149-55.Elley CR, Kerse N, Chondros P, Robinson E. Intraclass correlation coefficients from three cluster randomised controlled trials in primary and residential health care. Australian & New Zealand Journal of Public Health 2005;29:461-7.
12. Carey R, Lloyd R. Measuring Quality improvement in healthcare: A guide to statistical process control applications. Milwaukee, Wisconsin: American Society for Quality. Quality Press; 1995.
13. Taljaard M, Weijer C, Grimshaw JM, Eccles MP, the Ottawa Ethics of cluster Randomised Trials Consensus Group. The Ottawa Statement on the ethical design and conduct of cluster randomised trials: précis for researchers and research ethics committees. British Medical Journal 2013;346:f2838.

© The University of Auckland, 2013

15. Study Acknowledgement

STUDY ACKNOWLEDGMENT

I have read the protocol and agree that it contains all necessary details for carrying out the study as described. I will conduct this protocol as outlined therein and will make a reasonable effort to complete the study within the time designated.

I will provide copies of the protocol and access to all information to study personnel under my supervision. I will discuss this material with them to ensure that they are fully informed about the treatment and the study.

I understand that the study may be terminated or enrolment suspended at any time if it becomes necessary to protect the best interests of the study participants.

______________________________________ ________________

Investigator's printed name and signature Date

Address of study site:

© The University of Auckland, 2013

16. Appendix 1 – Terms of Reference

16.1 Advisory Group

The Advisory Group will consist of principal investigators, NIHI Director, PHO representatives, Heart Foundation representative, experts in Māori health, primary care, and laboratory testing and will be responsible for providing strategic guidance for the study including developing and maintaining the study design, approval of the protocol, statistical analysis, presentation and publication of results. The Advisory Group will be consulted by email as required during the study to review problems and issues raised by the Study Management Committee. If a formal meeting is required then members may attend in person or participate via conference call.

16.2 Study Management Committee

The Study Management Committee will be responsible for the daily operation of the study, and will develop study materials, deal with study problems, recruitment, and logistical issues. Meetings will be held regularly while the study is in development, then as required when the study is underway. Members who live outside of Auckland may attend the meetings, or participate via conference call.

© The University of Auckland, 2013

17. Appendix 2 – Study information sheet

Health Professional Information Sheet
Evaluating a Point-of-Care device in Heart Healthcare - EPOCH study

Introduction
Cardiovascular disease (CVD) risk assessment includes measurement of lipid and diabetes risk factors. However, current standard practice of laboratory-based testing may result in delays, or lack of completion, of these assessments.

The EPOCH study has been developed by the University of Auckland in collaboration with Mania and Te Tai Tokerau PHOs, Enigma Publishing Ltd, and the National Heart Foundation.

About the research
We are undertaking a research study to better understand if providing point-of-care (POC) testing in real time for lipids (TC/HDL) and diabetes (HbA1c) in a GP practice results in a higher rate of CVD risk assessment completion than standard laboratory-based testing.

This research has several main aims:
1. To evaluate the impact of POC testing for lipids and diabetes on the frequency of completed and incomplete CVD risk assessments in the eligible population.
2. To analyse the impact of POC testing on CVD risk assessments by age, sex, deprivation quintile, for Māori and non-Māori, diabetes, smoking status, and previous CVD risk score.
.
3. To evaluate the impact of POC testing on the average duration of CVD risk assessment.
4. To evaluate whether the impact of POC testing is the same in urban areas (e.g. Whangarei City) and rural areas; and in small and large GP practices.
5. To evaluate the acceptability, feasibility, and practice impact of a POC device in general practice.
6. To undertake a cost analysis.

If you agree to take part in the study, your practice will be randomly allocated to receive a Cobas b 101 POC device or to continue with laboratory testing.

Full training on using the device will be provided by the manufacturer (Roche Diagnostics NZ). The study investigators will collect de-identified data on PREDICT CVD risk assessments performed by all participating practices over a 3-6 month period. The data collected and transferred to the PREDICT database via the CVD risk assessment will be unidentifiable by anyone other than health professionals involved in the delivery of care to your patients.

Benefits and safety
The benefits to you and your patients are the opportunities to assess CVD risk using real time lipid and diabetes results in a single consultation. The Cobas b 101 device is currently available in New Zealand for use in testing patients for lipids and diabetes.

Participation
Your de-identified patient data will only contribute to the research if you have provided written agreement to participate in the study. If you do not agree to participate this will in no way affect your future relationships with the investigators or the University or the PHO.

Confidentiality

© The University of Auckland, 2013

Patient data collected from the POC device will be stored on your patient management system and the PREDICT server as part of usual clinical management. With permission from each participating practice and the PHO, de-identified data, using encrypted NHIs can be extracted for research purposes. All data will be treated as confidential and no material that could personally identify you or your patients will be used in any research reports. We may publish aggregated results in medical journals and present findings to health care conferences and CME meetings.

This study has received ethical approval from the Northern B Health and Disability Ethics Committee. Committee No: 13/NTB/79
Please feel free to contact the University of Auckland EPOCH Team or your PHO facilitator if you have any questions about the research

Ph 09 373 7599 ext 82358 or 84765

© The University of Auckland, 2013

18. Appendix 3 – Cobas b 101 medical device

The cobas b 101 system is intended for professional use in a clinical laboratory setting or point-of-care (PoC) locations.

HbA1c

The cobas b 101 system is an in vitro diagnostic test system designed to quantitatively determine the percent hemoglobin A1c (DCCT/NGSP) and mmol/mol (IFCC) in human capillary and venous whole blood by photometric transmission measurement. An estimated average glucose level (eAG) is calculated by the cobas b 101 system.

Lipid panel

The cobas b 101 system is an in vitro diagnostic test system designed to quantitatively determine total cholesterol (CHOL), high-density lipoprotein cholesterol (HDL), and triglycerides (TG) in human capillary and venous whole blood and plasma by photometric transmission measurement. A calculated value for low-density lipoprotein (LDL), Non-HDL and the CHOL/HDL ratio is provided by the cobas b 101 system.

Multicenter Evaluation

An evaluation of the cobas b 101 was carried out at two ISO-certified clinical laboratory sites: Barcelona, Spain and Zurich, Switzerland. The main objective of the performance evaluation was to confirm the analytical performance of the system in the hands of healthcare professionals in a point-of-care environment.

135 patients (71 male, 64 female) were recruited with an HbA1c range from 4.1 % to 13.6 % HbA1c. 160 patients (84 male, 76 female) were recruited with lipid panel ranges: for TC from 1.9 – 12.63 mmol/L, for TG from 0.52 – 6.7 mmol/L and for HDL from 0.47 – 2.44 mmol/L.

Key conclusions

All measurements evaluated with both the HbA1c disc and the lipid panel disc on the cobas b 101 system met the pre-defined acceptance criteria for lot-to-lot reproducibility, precision and method comparison to the reference system (see Performance Evaluation Results). The acceptance criteria for HbA1c were defined according to the National Glycohemoglobin Standardization Program (NGSP) guidelines as written prior to September 2012, as per the dates when the evaluation was carried out. Additional acceptance criteria were defined according to the Clinical and Laboratory Standards Institute (CLSI) guidelines and the National Cholesterol Education Program (NCEP) guidelines.

All sites confirmed the usability and practicability of the cobas b 101 system and rated the system as convenient for use in a point-of-care environment.

© The University of Auckland, 2013

19. Appendix 4 - Cobas b 101 quality assurance

Regulatory status

EU approved and marketed. New Zealand marketed (no approval required, class 2 IVD).

Validation testing

Two laboratories tested a range of samples for HbA1c (135 patients) and lipids (160 patients) using a cobas c 501 analyser as reference. All measurements met predefined acceptance criteria.

Canterbury Health Limited (CHL) 3 stage testing. Results from the first two stages demonstrate that the cobas b 101 correlates well with laboratory analysers on whole blood samples. Further results are expected on capillary blood samples. The correlation data was obtained using an Abbott Diagnostics analyser as the reference analyser.

Counties Manukau District Health Board (CMDHB) and LabTests are due to start a parallel validation study on 50 patients with capillary samples tested on the cobas b 101 and venous samples tested on the cobas b 101 and the LabTests analyser.

This correlation data will provide objective evidence that the test results obtained from the cobas b 101 are accurate and precise.

EPOCH quality assurance

The introduction of any point-of-care testing service needs to be supported with a comprehensive staff competency, quality assurance and risk management programme. Comprehensive staff competency and ongoing Internal Quality Control (IQC) and External Quality Assurance (EQA) programmes are required to support the point-of-care service in a field environment. The programme outlined below is based on the requirements of the ISO 22870: 2006(E) standard and the NZ Best Practice Guidelines for Point-of-care Testing. Both CHL and Northland District Health Board (NDHB) are accredited by International Accreditation New Zealand against the medical point-of-care testing standard ISO 22870: 2006(E) for point-of-care testing.

(1) Staff Acceptance and Competency

Roche Diagnostics NZ Ltd (Roche) will train and certify practice staff ('operators'). Training will be approximately 1.5-2 hours long and includes a presentation on patient testing, QC testing, and maintenance. All operators will be shown the best technique for testing and be required to perform a finger prick test and complete a short written test to pass. Training will emphasise that operators need to:

(a) be willing to perform this testing on an ongoing basis throughout the trial;

(b) have the time to actually do the testing on an as needed basis on top of other duties;

(c) adhere to correct capillary sampling and test methodology at all times. This will ensure that consistent and accurate results can be obtained over a long period;

(d) be aware that they will be responsible for carrying out any routine maintenance and quality control checks in order to ensure accurate test results at all times.

© The University of Auckland, 2013

It is inevitable that staff members may change over this time period so new operators may need to be trained. A train the trainer system will be used for this with the assistance of Roche where necessary. The designated trainer must be able to carry out on site training of new staff correctly and consistently.

If an abnormal or unexpected result (for lipids or HbA1c) occurs during operator training, the operator will be advised to see their general practitioner for clinical follow-up which may include laboratory testing at the GPs usual laboratory.

(2) Internal Quality Control

New batches of cartridges will undergo acceptance testing to ensure that each batch works correctly prior to use.

IQC testing will be performed monthly by the practices. This will ensure the ongoing stability of the kits and the analysers. The data will also provide valuable statistical information across the group.

Additional IQC tests need to be performed in the event of the following: the cobas b 101 device is dropped or damaged or subject to a power surge; cobas b 101 device malfunction; the test results obtained for a patient do not correlate with the patient's clinical state.

(3) External Quality Assurance and Inter-Laboratory Comparison Programmes (ILCP)

EQA will include a reference cobas b 101 situated centrally at NDHB participating in the accredited HbA1c EQA programme run by Waikato Hospital or monthly correlation studies with the local laboratory (ILCP). A whole blood comparison lipid programme will be developed in house by NDHB.

(4) Review of IQC and EQA Data

Geoff Herd (Point-of-Care Testing Coordinator, NDHB) will provide oversight for the IQC and EQA programmes. Clinical issues will be referred to the EPOCH Study Management Committee and/or Advisory Group.

(5) Troubleshooting

There may be problems with the cobas b 101 and the cartridges for time to time and so telephone support by Roche Diagnostics will be available. Because of the local geography and relative lack of courier systems a back-up analyser will be placed in NDHB so that it can be easily shipped to any site which is having problems. This device will also be used for IQC, as a reference EQA machine and troubleshooting.

© The University of Auckland, 2013

20. Appendix 5 – Proposed Timeline

The timeline for the study is:

**Year**	**2013**			**2014**				**2015**	
**Activity**	**Q2**	**Q3**	**Q4**	**Q1**	**Q2**	**Q3**	**Q4**	**Q1**	**Q2**
Study start up (study materials, ethics, staff)	x								
Participant recruitment		x							
Study follow-up			x	x	x	x	x	x	
Data analysis									x
Preparation of main report									x

Key milestones

**Date**	**Milestone**
May 2013	Ethics submitted
June 2013	Ethics approval
July 2013	Recruit practices
August 2013	Start of study
August 2013	Study halted due to manufacturing problems cobas b 1010 discs
May 2014	Study recommenced after re-validation cobas discs, retraining practices and Ethics approval
June 2015	Estimated end of study
September 2015	Analysis complete.
November 2015	Paper submitted.

© The University of Auckland, 2013

21. Appendix 6 – Summary of Protocol Amendments

Page	Section heading	Amendment
All	Header	From: Version 3<br>To: Version 4<br>Updated version number
All	Footer	From: 5/8/2014<br>To: 30/3/2015<br>Updated date
3	Advisory Group Members	From: Dr Susan Wells<br>To: A/Professor Susan Wells<br>From: Assoc Prof Chris Bullen<br>To: Professor Chris Bullen<br>Updated details
4/5	Study Management Committee Members	From: Dr Susan Wells<br>To: A/Professor Susan Wells<br>From: Assoc Prof Chris Bullen<br>To: Professor Chris Bullen<br>From: Taina von Blaramberg<br>To Angela Wadham<br>Updated Committee Member details<br>Angela Wadham new Project Manager replacing Taina von Blaramberg
7	NIHI Signatory	From: Dr Susan Wells<br>To: A/Professor Susan Wells<br>From: Assoc Prof Chris Bullen<br>To: Professor Chris Bullen
7	Revision Chronology	Version 4 – 30 March 2015 – Amendment 3<br>New version information added
10	Investigators and study centres	From:Dr Sue Wells, Dr Natasha Rafter, Assoc Prof Chris Bullen, The University of Auckland.<br>To: A/Prof Sue Wells, Dr Natasha Rafter, Prof Chris Bullen, The University of Auckland.
12	Study Plan Schematic	Added: End of study practice questionnaire to Laboratory group arm
18	6 Study Design<br>Para 3	From: The eligible population is comprised of patients enrolled in these practices who are aged between 35-79 years, who meet age, sex and ethnicity guideline criteria for CVD risk assessment<br><br>To: The eligible population is comprised of patients enrolled in these practices who are aged between 35-79 years, who as determined by their GP, meet age, sex and ethnicity guideline criteria for CVD risk assessment
18	6.1 Inclusion Criteria	From: Patients will be included in the eligible study population if they<br>• are aged at least 35 years and less than 80 years old, and

© The University of Auckland, 2013

		• meet national age, sex and ethnicity guideline criteria for CVD risk assessment (Table 1) and repeat assessment recommendations (Table 2)<br><br>To: Patients will be included in the eligible study population if<br>• they are aged at least 35 years and less than 80 years old, and<br>• as determined by their GP, they meet national age, sex and ethnicity guideline criteria for CVD risk assessment (Table 1) and repeat assessment recommendations (Table 2)
23	Table 1: Details of follow-up	Delete column mid study questionnaire
25	7.2.4 Cost Analysis	From: A cost analysis will be performed for both groups based on practice consultations and testing for lipids and diabetes (laboratory or POC). Data on numbers of practice consultations, laboratory and POC device tests will be sourced from PREDICT. Cost data will be obtained from the PHOs, local laboratories and Roche Diagnostics. Qualitative information on other practice resource use will be collected through the practice follow-up.<br>To: A cost analysis will be performed for both groups based on practice consultations and testing for lipids and diabetes (laboratory or POC) from a payer perspective. Data on numbers of practice consultations, laboratory and POC device tests will be sourced from PREDICT. Unit cost data will be obtained from the PHOs, local laboratories and Roche Diagnostics. Qualitative information on other practice resource use will be collected through the practice follow-up. Practice staff costs will be assessed by asking practices about the amount of nurse and GP time required under POC and laboratory testing.
43	Appendix 5 Proposed Timeline	Table updated
43	Appendix 5 Key milestones	From:<br>November 2014 Estimated end of study<br>March 2015 Analysis complete<br>May 2015 Paper submitted<br>To:<br>June 2015 Estimated end of study<br>September 2015 Analysis complete<br>November 2015 Paper submitted<br><br>Amended to reflect changes to timeline.

© The University of Auckland, 2013
